# Drug loaded cerium oxide nanozymes prevent radiation-Induced cataracts via suppressing the cGAS-STING pathway

**DOI:** 10.1186/s12951-025-03706-2

**Published:** 2025-10-10

**Authors:** Qifang Chen, Peilin Gu, Hongwei Li, Xuemei Liu, Ting Liu, Qin Ouyang, Dong Liu, Chongyi Li

**Affiliations:** 1 Department of Ophthalmology, Daping Hospital, Army Medical University, Chongqing, 400042 China; 2 Department of Pharmaceutical Chemistry, Army Medical University, Chongqing, 400038 China; 3 State Key Laboratory of Ophthalmology, Zhongshan Ophthalmic Center, Sun Yat-sen University, Guangzhou, 510060 China

**Keywords:** Radiation-induced cataracts, Nanozymes, CGAS-STING pathway, Antioxidative, Anti-inflammatory

## Abstract

Radiation-induced cataracts (RIC), a significant cause of blindness, arise from oxidative damage and inflammation in the lens epithelium, and there has been a lack of effective targeted therapeutic drugs in clinic. Here, we proposed and developed a novel approach for prevention and treatment of RIC via multifunctional nanozymes. We first demonstrated activation of the cGAS-STING pathway can drive mitochondrial oxidative stress and cytosolic double-stranded DNA (dsDNA) accumulation in ionizing radiation (IR)-exposed lens epithelial cells (LECs). Accordingly, a nanozyme-based drug delivery system, namely porous CeO_2_ loaded with STING inhibitors H-151 (CeO_2_@H-151), was developed for RIC prevention and treatment. Importantly, CeO_2_@H-151 nanozymes not only decreased reactive oxygen species (ROS) production induced by mitochondrial oxidative injury, but also reduced inflammation caused by increased dsDNA. This can be attributed to the synergistic effect of antioxidant, anti-inflammation and protein homeostasis-preserving of CeO_2_@H-151 nanozymes. It also revealed CeO_2_@H-151 nanozymes exerted protein homeostasis preservation through H-151 bound to the Cys172 oxidative modification site of PRDX2. This work provides a synergistic nanotherapeutic strategy targeting the cGAS-STING pathway, offering a promising approach for preventing and treating RIC and even other oxidative-inflammatory disorders.

**Graphical Abstract:**

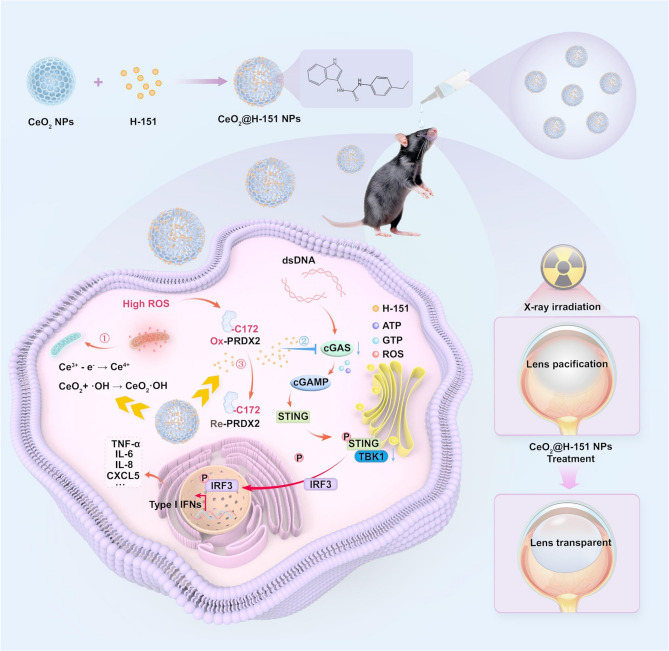

**Supplementary Information:**

The online version contains supplementary material available at 10.1186/s12951-025-03706-2.

## Introduction

Cataracts are the leading cause of blindness worldwide, accounting for approximately 46% of blindness globally [1]. Among them, radiation-induced cataract (RIC) that caused by ionizing radiation (IR) exposure, such as X-rays, gamma rays and neutron beams is also a serious concern for vision health [2]. Previous studies have demonstrated that it involves multiple pathological processes like oxidative stress, DNA damage, mitochondrial dysfunction, inflammation, cytokine signaling and protein oxidative modification in lens epithelial cells (LECs) after IR exposure, resulting in cellular senescence, apoptosis and ultimately leading to lens opacity [3–5]. Specially, IR-induced DNA damage and inflammatory responses could activate the cGAS-STING pathway, where cytosolic DNA sensors like cGAS detect nuclear/mitochondrial DNA leaks, generating cGAMP to stimulate STING [6–8]. Recent studies have shown that genome instability induced by IR exposure led to the accumulation of micronuclear DNA, which activated cGAS-mediated inflammatory response and secretion of CCL5 [9]. More evidences indicated that targeting the cGAS–STING pathway could modulate the inflammatory response induced by IR, providing new insights for the prevention of IR-induced cataracts [10]. Here, we first demonstrated that activation of the cGAS-STING pathway can drive mitochondrial oxidative stress and cytosolic double-stranded DNA (dsDNA) accumulation in IR-exposed LECs.

Considering the important role of cGAS-STING pathway in the formation of IRC, STING inhibitors may be a promising therapeutic strategy. However, the hydrophobic nature of STING inhibitors significantly limits their ocular bioavailability [11, 12]. In recent years, nanotechnology-based drug delivery systems (DDS) have gained great attention in ophthalmic diseases due to their nano-scale size, high drug-loading capacity, and ability to overcome ocular barriers (e.g., corneal epithelium, blood-retinal barrier) [13–16]. For example, nanosuspensions, as a drug delivery system, are composed of nanoscale colloidal dispersions to enable hydrophobic drugs to disperse uniformly in aqueous media, thereby improving bioavailability and corneal penetration (e.g., brinzolamide nanosuspensions for glaucoma treatment) [17, 18]. Liposomes have been widely used to deliver mRNA therapeutics, such as COVID-19 vaccines and gene therapy for inherited retinal diseases [19, 20]. Nanomicelles composed of amphiphilic block copolymers are often used to deliver hydrophobic therapeutic compounds due to their enhanced stability and sustained drug release properties, as demonstrated in the delivery of dexamethasone for diabetic macular edema [21].

Nanozymes have emerged as highly anticipated therapeutic agents due to their biocatalytic activity and reactive oxygen species (ROS)-scavenging capabilities [22, 23]. Among them, nano-cerium oxide (CeO_2_) is a multi-enzyme mimetic with superoxide dismutase (SOD) and catalase (CAT) activities, as well as the ability to scavenge hydroxyl radicals, which has been widely used in neurodegenerative diseases, wound healing, and ocular disorders such as dry eye disease (DED) and age-related macular degeneration (AMD) [24–27]. Recently, eye drops containing CeO_2_ nanoparticles have exhibited long-term anti-inflammatory and antioxidant properties, along with prolonged ocular surface retention time, good ocular tolerance and biosafety, achieving excellent therapeutic effect in murine models of dry eye and corneal injury [28].

**Fig. 1:**
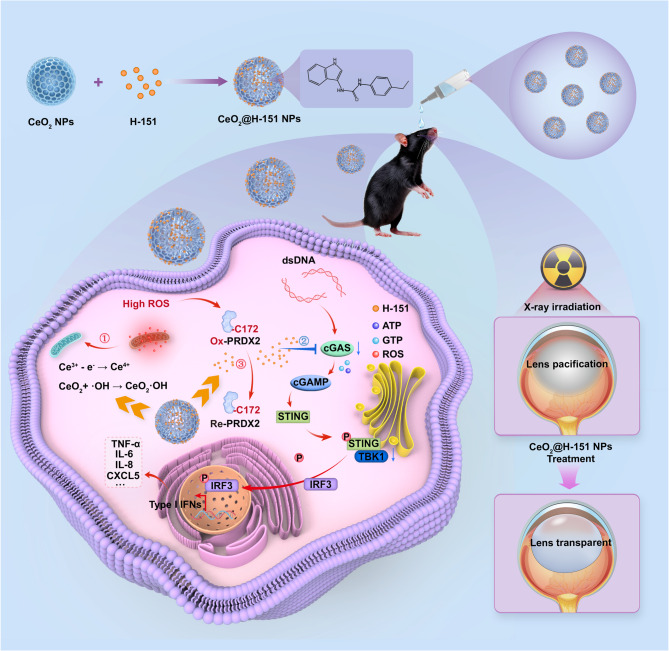
Schematic diagram of CeO₂@H-151 nanozymes in preventing radiation-induced cataract and the mechanisms. After topical administration, the CeO₂@H-151 nanozymes can restore lens redox homeostasis via Ce³⁺/Ce⁴⁺-mediated ROS scavenging, suppress the cGAS-STING pathway through controlled release of H-151 to inhibit inflammatory responses, and reduce oxidative modification of PRDX2 that is the peroxidase responds to oxidative stress and maintains intracellular redox balance at the Cys172 site induced by IR, thereby preventing lens opacity

In this study, a nanozyme-based DDS was designed to inhibit the cGAS-STING pathway for prevention and treatment of IR-induced cataracts. We constructed hollow mesoporous CeO_2_ loaded with STING inhibitors H-151 (CeO_2_@H-151 nanozymes) to simultaneously reduce mitochondrial ROS levels, scavenge cytosolic dsDNA leaked from damaged mitochondria, suppress inflammation, and decrease PRDX2 protein oxidative modification. This synergistic effect could inhibit the overactivation of the cGAS-STING pathway, thereby preventing LECs damage and subsequent lens opacity (Fig. 1). The remarkable therapeutic efficacy of CeO_2_@H-151 nanozymes in vitro and in vivo suggests that targeting the cGAS-STING to exert antioxidant and anti-inflammatory is an efficient strategy for preventing IR-induced cataracts that currently lacking effective pharmacological interventions. Similarly, previous studies have highlighted the role of mitochondrial DNA (mtDNA)-triggered cGAS-STING activation in age-related cataracts. Cai et al. showed that CeO_2_ nanoparticles could safely reduce oxidative stress in retinal degeneration models without adverse effects [29]. These findings strongly support the suitability of CeO_2_@H-151 as topical eye drops for preventing and treating IR-induced cataracts, offering a non-invasive, long-term therapeutic strategy with dual ROS-scavenging and anti-inflammatory capabilities. Furthermore, CeO_2_@H-151 nanozymes, as a multifunctional and biocompatible scavenging system for ROS and pro-inflammatory cytokines, may have promising translational potential for clinical application, particularly for radiation-exposed population.

## Materials and methods

### Materials

Hollow mesopores CeO_2_ nanoparticles were purchased from Jike Biotechnology Co., Ltd (Nanjing, China). Cy5@CeO_2_@H-151 nanoparticles were purchased from Ruixi Biological Technology Co., Ltd (Xi’an, China). STING inhibitors (H-151) and Cy5 probes were all obtained from Bocai Biotechnology Co., Ltd (Chongqing, China). Superoxide dismutase (SOD), catalase (CAT), Glutathione (GSH), malondialdehyde (MDA) and adenosine triphosphate (ATP) assay kits were all acquired from Solarbio Science &Technology Co., Ltd (Beijing, China). JC-1 mitochondrial membrane potential (MMP, △Ψm) assay kit was purchased from Bioss Biological Technology Co., Ltd (Beijing, China). 2,7-Dichloro-fluorescein diacetate (DCFH-DA) was purchased from Beyotime Biotechnology Co., Ltd (Shanghai, China). H-151@cy5 probe was prepared from Outdo Biotech Co., Ltd (Shanghai, China). ABplex Human 13-Plex Custom Panel and Rhodamine Phalloidin were from ABclonal Biotechnology Co., Ltd. (Wuhan, China).

### Preparation of CeO_2_@H-151 nanozymes

For synthesis of CeO_2_@H-151 nanozymes, 0.2 mg of a H-151 solution prepared in 144 µL of DMSO was dropped into CeO_2_ nanoparticle solution slowly under continuous stirring at 1200 rpm for 6 h. Further, the mixture was transferred to a dialysis bag (MWCO: 3.0 KD) and purified for three times with deionized water for another 24 h, which was stored at 4 °C to next usage.

### Characterization of CeO_2_@H-151 nanozymes

The morphology observation and elemental characterization of CeO_2_@H-151 nanozymes was performed by high-resolution transmission electron microscopy (HRTEM, FEI Tecnai G2 F30, Oxford XPLORE). The hydrodynamic size and zeta potential of CeO_2_@H-151 nanozymes were measured by a dynamic light scattering (DLS) system (NanoZS; Malvern Instruments, Worcestershire, UK). The absorbance changes were detected by an ultraviolet-visible spectroscopy (UV-vis, UV-1800, Shimadzu, Japan). The X-ray photoelectron spectra (XPS) were examined by an ESCA system (PHI 1600, PerkinElmer). Fourier transform infrared (FTIR, Nicolet IS10, USA) spectrometer was performed to analyze the presence of functional groups and characteristic bands in the CeO_2_@H-151 nanozymes in the range of 800–3700 cm^−1^. Fully automatic surface area and porosity analyzer (TriStar II Plus, Micromeritics ASAP 2460), which recorded physical adsorption isotherms at 77 K and calculated the specific surface area using the Brunauer-Emmett-Teller (BET) method. It employed the Barrett-Joyner-Halenda (BJH) pore analysis method to derive total pore volume, average pore diameter, and pore size distribution curve.

### In vitro release evaluation CeO_2_@H-151 nanozymes

High performance liquid chromatography (HPLC) was performed to investigate the release behavior of H-151 from suspension and CeO_2_@H-151 nanozymes. The 500 µL of samples in the dialysis bags (MWCO: 3.0 KD) were placed into in 10 mL of phosphate buffer saline (PBS, pH = 7.0) under stirring at 37 °C, which removed from the release medium at 0.5, 1, 2, 5, 10, 12, 24, 36 and 48 h. Then, the removal solutions were determined using HPLC. We used a Waters 2489 HPLC system (Waters, USA) and a SunFire C18 column (150 mm×4.6 mm, 5 μm) liquid chromatography column to detect the release behaviors of H-151 in the CeO_2_@H-151 nanozymes. The mixture of methanol and water (70:30, volume/volume) was used as the mobile phase at room temperature, the flow rate was 0.8 mL/min, the injection volume was 10 µL, and the absorption wavelength was set at 254 nm. The standard curve of drug H-151 was established with drug concentration as the horizontal axis and the main peak area as the vertical axis. Next, the absorbance of the prepared CeO_2_@H-151 nanozymes was detected at a wavelength of 254 nm, and the content of H-151 in the CeO_2_@H-151 nanozymes was calculated as the target peak area.

### Cellular uptake CeO_2_@H-151 nanozymes in vitro

HLECB3 cells were inoculated in the confocal dishes for 12 h, which pre-treated with H-151@Cy5 probe and CeO_2_@H-151@Cy5 nanoparticles for 0, 2, 4, 6 h. After that, cellular uptake via red fluorescence intensity was observed using confocal laser scanning microscope (CLSM) and flow cytometry (FCM, BD FACS Melody™).

### Intracellular ROS and mitochondrial membrane potential detection

16 Gy of X-rays radiation of HLECB3 cells was applied to construct an oxidative stress model. A DCFH-DA probe was performed to detect the intracellular ROS levels. After treatment with H-151, CeO_2_ and CeO_2_@H-151 nanozymes, we used CLSM and FCM to analyze the green fluorescence intensity in the LECs. Similarly, MMP was measured by incubation with JC-1 probe in PBS for 30 min at 37 °C. Then, the ratio of red/green fluorescence in the LECs treated with H-151, CeO_2_ and CeO_2_@H-151 nanozymes was determined by CLSM and FCM.

### Western blot and real-time PCR analysis

The expression levels of anti-p-cGAS, anti-cGAS, anti-p-STING, anti-STING, anti-p-TBK1, anti-TBK1, anti-p-IRF3, anti-IRF3, anti-β-actin, anti-GAPDH purchased from ABclonal Biotechnology Co., Ltd. (Wuhan, China) were determined by a ChemiDocTM Touch imaging system (ChemiDoc Touch). We used the Simply P Total RNA Extraction Kit (BioFlux) and reverse transcribed with a PrimeScript RTPCR kit (Takara Biotechnology) to extract and detect cellular total RNA concentrations according to the manufacturer’s instructions. Then, the real-time PCR analysis was performed using a standard SYBR Green PCR kit (Roche). The 2^−△△Ct^ method was used to calculate the relative expression levels.

### RNA sequencing assay and functional classification

First, we inoculated 5*10^6^ cells/plate of LECs and cultured for 24 h. The LECs were treated with H-151, CeO_2_ and CeO_2_@H-151 nanozymes after 16 Gy X-rays radiation for another 24 h. Then, we collected these cells added into 1mL of Trizol reagent to analyze RNA sequence in Outdo Biotech Co., Ltd (Shanghai, China) on NovaSeq 6000 platform (Illumina). The differential expression genes (DEGs) were performed using the DESqe R package (1.20.0) with P-value < 0.05 and |log2foldchange| >1. A signaling pathway enrichment analysis was performed using kyoto encyclopedia of genes and genomes (KEGG) with the clusterProfiler software (3.4.4).

### Redox proteomics

The mice lens samples of IR, IR/H-151 and IR/CeO_2_@H-151 groups (*n* = 2) were dried, desalted, and concentrated under vacuum. Peptides were dissolved in 10 µL of 0.1% formic acid (FA) for subsequent LC-MS/MS analysis. LC-MS/MS were performed on a Orbitrap Astral mass spectrometer coupled with Vanquish Neo UHPLC system (Thermo Fisher Scientific). The DIA MS data were analyzed using Spectronaut 18 (Biognosys AG, Switzerland). MS data were searched against the UniProtKB reviewed (Swiss-prot) database (Homo sapiens (species), 20427 total entries, downloaded 10/2023. All sample preparation, LC-MS analysis and bioinformatics analysis were completed at Bioprofile Biotechnology Co., Ltd (Shanghai, China).

### Retention and distribution of CeO_2_@H-151 nanozymes in vivo

Male (3–5 weeks, around 18 g) C57BL/6J mice were purchased from the HFK Bio-Technology Co., LTD (Beijing, China), which fed in specific pathogen-free (SPF) condition in the Animal Centre of Daping Hospital (Chongqing, China). 10 µL of free Cy5 and CeO_2_@H-151@Cy5 nanoparticles (same amount of Cy5) were topically instilled after the mice were anesthetized (*n* = 3) for 14 days. To evaluate the retention and distribution of Cy5 and CeO_2_@H-151@Cy5 nanoparticles in the eye, the mice were sacrificed and the entire eyes were collected. And the eyes sections were observed by hematoxylin-eosin (HE) staining to evaluate the safety of CeO_2_@H-151@Cy5 nanoparticles. Besides, the red fluorescence in the sections of Cy5 and CeO_2_@H-151@Cy5 nanoparticles treatments was observed by CLSM to evaluate the retention and distribution of CeO_2_@H-151 nanozymes.

### IR-induced cataract mouse model establishment and evaluation of CeO_2_@H-151 nanozymes prevention capacity in vivo

16 Gy X-rays radiation was adopted to induce the cataract mouse model (*n* = 10). 10 µL of H-151, CeO_2_ and CeO_2_@H-151 nanozymes were treated with topical eye drops twice per day to evaluate the effects after 16 Gy X-rays radiation for once. After 35 days of prevention, we used the slit lamp to observe the lens opacity. After the mice were sacrificed, the lens tissue was extracted to perform the Total oxidation modification sequencing in Bioprofile Co., LTD (Shanghai, China). The mechanism of the prevention capacity of H-151 and CeO_2_@H-151 nanozymes in IR-induced cataracts was explained from the perspective of preventing the protein oxidation modification.

### Molecular Docking

The structure of the protein PRDX2 were obtained from AlphaFold Protein Structure Database (Uniprot ID: P32119, https://www.alphafold.ebi.ac.uk/). And then, the structures were prepared by SYBYL-X 2.0 using the Powell method under AMBER7 FF99 force field and AMBER charges. The structure of the compound H-151 was downloaded from PubChem Database (https://pubchem.ncbi.nlm.nih.gov/, CAS: 941987-60-6). Then, the docking study of PRDX2 and H-151 was performed using HelixFold3 server (https://paddlehelix.baidu.com/app/all/helixfold3/forecast). The top binding pose predicted by HelixFold3 server was used to analyze the interactions between PRDX2 and H-151. The binding interactions were analyzed by PLIP web tool (https://plip-tool.biotec.tu-dresden.de/plip -web/plip/*)* and ligplot v2.2.5 and visualized using PyMOL V2.52.

### Statistical analysis

Comparisons between different groups were performed with a Student’s t-test (two-group) and one-way ANOVA in the multiple groups as appropriate using GraphPadPrism (ver. 9.0). Comparisons with multiple variables were determined by two-way ANOVA. Statistical data were expressed as means ± standard deviation (SD) as indicated. *P < 0.05, **P < 0.01, ***P < 0.001, ****P < 0.0001, and n.s. indicated no significance.

## Results and discussion

### Activation of the cGAS-STING pathway in LECs upon ionizing radiation (IR) exposure

**Fig. 2:**
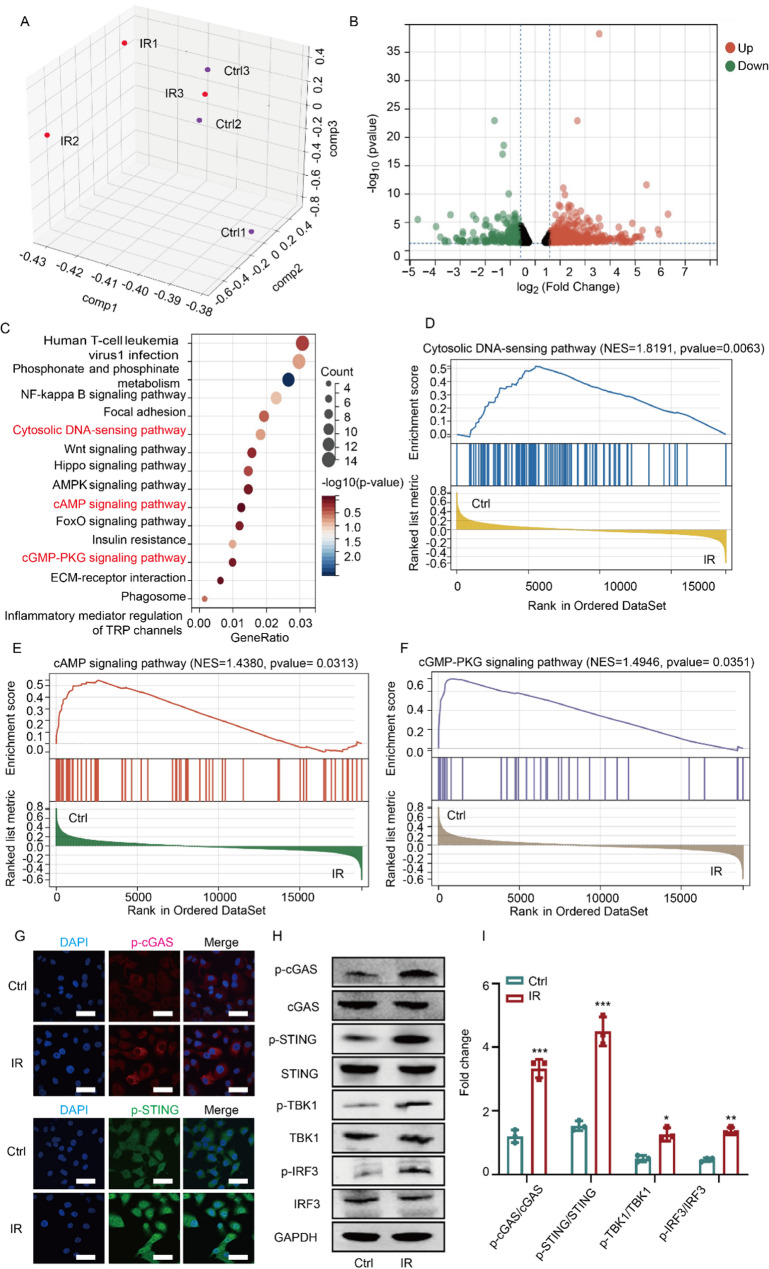
The cGAS-STING pathway was activated in LECs after IR. (**A**) PCA analysis of the control and IR groups (*n* = 3). (**B**) Volcano plot of gene expression in control and IR groups. (**C**) The KEGG function enrichment and GSEA (**D**-**F**) analysis after IR treatment. (**G**) IF staining of p-cGAS (red) and p-STING (green) in LECs after IR. Scale bar: 30 μm. (**H**) WB and quantitative analysis (**I**) of proteins involved in the cGAS-STING pathway (*n* = 3). *P < 0.05, **P < 0.01, ***P < 0.001

Radiation causes DNA fragmentation through direct damage and indirect oxidation, leading to abnormal cell migration and complex biochemical changes, which result in abnormal lens protein folding and disorganization of lens cell morphology [30]. The cGAS-STING pathway is an important intracellular immune response pathway, primarily activated by sensing DNA damage and foreign DNA, such as viral DNA, to trigger immune responses [31]. In the case of oxidative stress, oxidative damage May affect the activation of cGAS-STING pathway, thereby influencing immune responses and inflammation. To investigate the role of the cGAS-STING pathway in IR-induced lens injury, we established in vitro models by exposing LECs to 16 Gy X-ray irradiation. Principal component analysis (PCA) demonstrated clear segregation between irradiated and control groups (Fig. 2A), indicating a robust transcriptional reprogramming response. Differential gene expression analysis further identified a substantial number of upregulated genes (Fig. 2B), suggesting activation of key signaling cascades. Furthermore, Kyoto Encyclopedia of Genes and Genomes (KEGG) pathway enrichment and Gene Set Enrichment Analysis (GSEA) highlighted significant upregulation of the cytosolic DNA-sensing pathway, cAMP signaling, and cGMP-PKG signaling pathways (Fig. 2C–F), all of which are mechanistically linked to the cGAS-STING pathway. Based on these findings, we hypothesized that IR triggers aberrant cytosolic DNA sensing, leading to cGAS-STING-mediated inflammatory signaling.

As the cGAS-STING pathway is activated when STING detects cyclic GMP-AMP (cGAMP), a secondary messenger synthesized by cyclic GMP-AMP synthase (cGAS) [32, 33]. Upon binding cGAMP, STING undergoes a conformational change and translocates from the endoplasmic reticulum to the Golgi apparatus, where it recruits and phosphorylates TANK-binding kinase 1 (TBK1) [34]. This, in turn, phosphorylates interferon regulatory factor 3 (IRF3), facilitating its nuclear translocation and subsequent binding to interferon (IFN) gene promoters to drive inflammatory gene expression. Our Western blot and immunofluorescence analyses also validated this process. Notably, irradiation exposure obviously upregulated the phosphorylation levels of cGAS and STING compared to control groups (Fig. 2G), confirming the induction of the cGAS-STING signaling cascade. Besides, Fig. 2H-I exhibited that elevation of the phosphorylation levels of TBK1 and IRF3 after IR exposure. These findings demonstrated that IR triggers aberrant cGAS activity, leading to downstream STING pathway activation in LECs, which may contribute to radiation-induced lens opacity.

### Preparation and characterization of CeO_2_@H-151 nanozymes

**Fig. 3:**
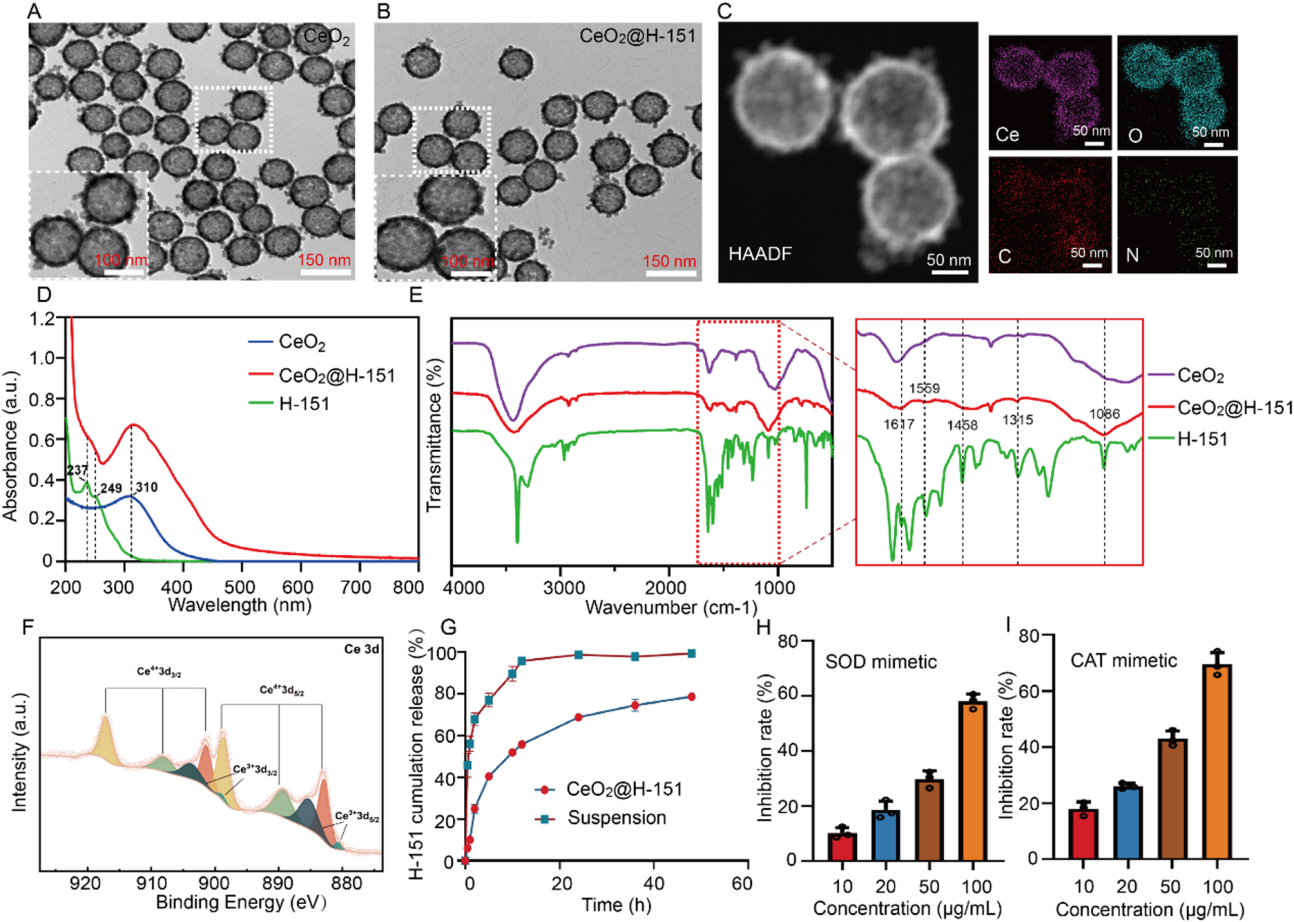
Characterization of the prepared CeO_2_@H-151 nanozymes. (**A**) Representative HRTEM images of CeO_2_ and CeO_2_@H-151 nanozymes (**B**). (**C**) HAADF images and EDS mapping of CeO_2_@H-151 nanozymes. (**D**) UV − vis spectrum of H-151, CeO_2_ and CeO_2_@H-151 nanozymes. (**E**) FTIR spectra of H-151, CeO_2_ and CeO_2_@H-151 nanozymes. (**F**) XPS analysis of CeO_2_@H-151 nanozymes. (**G**) In vitro drug release behavior of H-151 from CeO_2_@H-151 nanozymes and suspension (*n* = 3). (**H**) SOD mimics activities of CeO_2_@H-151 nanozymes (*n* = 3). (**I**) CAT mimics activities of CeO_2_@H-151 nanozymes (*n* = 3)

According to the key role of cGAS-STING pathway in the formation of RIC, a promising strategy for preventing and treating IR-induced lens damage lies in targeting the cGAS-STING pathway. In this study, we prepared hollow mesoporous CeO_2_ nanozymes loaded with STING inhibitor H-151 (CeO_2_@H-151), which exhibit antioxidant and anti-inflammatory activities as well as good biocompatible. Hollow mesoporous CeO_2_ nanozymes were synthesized via a modified templating method, followed by H-151 loading through surface adsorption, and the preparation process was illustrated in Fig. 1. As shown in Fig. 3A-B, High-resolution transmission electron microscopy (HRTEM) revealed that monodisperse spherical nanostructures of CeO_2_@H-151 (120 nm diameter), with High-angle annular dark-field scanning (HAADF-STEM) analysis confirming homogeneous H-151 distribution (Fig. 3C). Dynamic light scattering (DLS) showed hydrodynamic diameters of CeO_2_ (136.3 ± 2.1 nm) and CeO_2_@H-151 (142.5 ± 3.5 nm), with zeta potentials of −9.4 ± 1.5 mV and − 12.8 ± 1.2 mV, respectively (Fig. S1 A-B). Moreover, CeO_2_@H-151 exhibited excellent stability in other aqueous conditions, including phosphate-buffered saline (PBS), saline (0.9% sodium chloride (NaCl)), and serum-supplemented medium (SSM) via DLS analysis, suggesting CeO_2_@H-151 was suitable for subsequent in vitro and in vivo activity studies (Fig. S2). The presence of a characteristic absorption peak at ≈ 237 nm in the UV–vis absorbance spectrum confirmed the successful loading of H-151 into the CeO_2_ nanoparticles (Fig. 3D). As shown in Fig. 3E, the characteristic absorption peaks of the H-151 molecular structure were clearly observed in the FTIR spectrum. The absorption peak at 1617 cm⁻¹ corresponded to the stretching vibration of mononuclear aromatic C = C bonds, while the peaks at 1559 cm⁻¹ and 1458 cm⁻¹ were attributed to C = C stretching vibrations in the pyrrole and benzene rings, respectively. The peak at 1086 cm⁻¹ was assigned to the C-N stretching vibration in the pyrrole ring. Additionally, the presence of three distinct peaks at 3425 cm⁻¹, 1559 cm⁻¹, and 1315 cm⁻¹ confirmed the existence of secondary amide groups in the molecular structure. X-ray photoelectron spectroscopy (XPS) analysis (Fig. 3F and Fig. S3) confirmed that the CeO₂@H-151 mainly contained four elements (Ce, C, N, O), with the “N” element entirely originating from the encapsulated H-151. Separately, the presence of both Ce³⁺ and Ce⁴⁺ oxidation stated on the CeO_2_@H-151 surface, with a Ce³⁺ content of 23.38%. The characteristic peaks for Ce⁴⁺ were observed at 882.85 eV, 889.33 eV, and 898.65 eV (3d₅/₂) and 901.38 eV, 908.08 eV, and 917.08 eV (3d₃/₂), while Ce³⁺ peaks appeared at 880.66 eV and 885.29 eV (3d₅/₂) and 895.6 eV and 904.25 eV (3d₃/₂). The binding energy of “C” element at 284.8 eV corresponded to C-C, with a proportion of 53.49%; at 286.26 eV corresponded to C-O/C-N, with a proportion of 32.87%; and at 288.74 eV corresponded to C = O, with a proportion of 13.64%. The binding energy of “O” element at 530.06 eV corresponded to Lattice-O, with a proportion of 31.35%; at 532.49 eV corresponded to Vacancy-O, with a proportion of 68.65%. The binding energy of “N” element at 400.04 eV corresponds to C-N, with a proportion of 100%. This mixed-valence state enables the regenerative redox cycle that is crucial for the nanozyme’s reactive oxygen species (ROS) scavenging capability [35, 36].

To investigate the drug loading and in vitro release characteristics, high performance liquid chromatography (HPLC) analysis was performed, and the results revealed that CeO_2_@H-151 exhibited an encapsulation efficiency (EE %) of 47.4% ± 1.1% with a drug loading (DL %) of 4.5% ± 0.3% for H-151. As illustrated in Fig. 3G and Supplementary Fig. S4, the in vitro release profile of free H-151 suspension exhibited rapid and near-complete drug release, achieving 95.8% cumulative release within 12 h. In contrast, the CeO_2_@H-151 nanozymes demonstrated a controlled release kinetics characterized by an initial burst release phase, with 40.6% of the encapsulated H-151 released within the first 5 h, then followed by a sustained release phase mediated by gradual diffusion from the nanozyme matrix. This sustained release profile was advantageous for ocular treatment. Changes in the mesopore size and specific surface area of CeO_2_ before and after drug loading were compared using N_2_ adsorption/desorption isotherms to assess the drug loading efficiency. As shown in Fig. S5A-B, the CeO_2_ curve exhibited a type IV isotherm with an H_2_-type hysteresis loop, indicating the characteristic hollow mesoporous structure of CeO_2_. In contrast, the CeO_2_@H-151 curve displayed a type II isotherm, characteristic of physical adsorption by non-porous materials. Furthermore, the calculated specific surface area of CeO_2_ decreased from 24.7 m² g⁻¹ to 5.9 m² g⁻¹ after drug loading (CeO_2_@H-151), and the mesopores of CeO_2_ decreased from 13.2 nm to 6.4 nm (CeO_2_@H-151). These changes demonstrated that the H-151 occupied the majority of the mesopore within CeO_2_, achieving efficient drug loading. The hollow mesoporous CeO_2_ nanozymes demonstrated remarkable enzymatic catalytic activity, attributable to their unique structural and electronic properties including maximized exposure of active catalytic sites, high Ce³⁺ content with abundant oxygen vacancies, and efficient Ce⁴⁺/Ce³⁺ redox cycling. The CeO_2_@H-151 efficiently eliminated the O_2_^•−^ in a dose-dependent Manner, and even scavenged 58% of O_2_^•−^ at the concentration of 100 µg/mL, indicating the superoxide dismutase (SOD)-like catalytic activity (Fig. 3H). Besides, the catalase (CAT, which catalyzes the reduction of H_2_O_2_ to water and molecular oxygen) assay kit was used to test its CAT-mimicking property (Fig. 3I). Similarly, CeO_2_@H-151 exhibited dramatically higher H_2_O_2_ scavenging ability, confirming the good antioxidant activity (Fig. 3H). These results indicated that CeO_2_@H-151 possessed multi-enzymatic activities for scavenging both O_2_^•−^ and H_2_O_2_.

### Improved cellular uptake and corneal penetration

#### In vitro biocompatibility and cellular uptake of CeO_2_@H-151 nanozymes

**Fig. 4:**
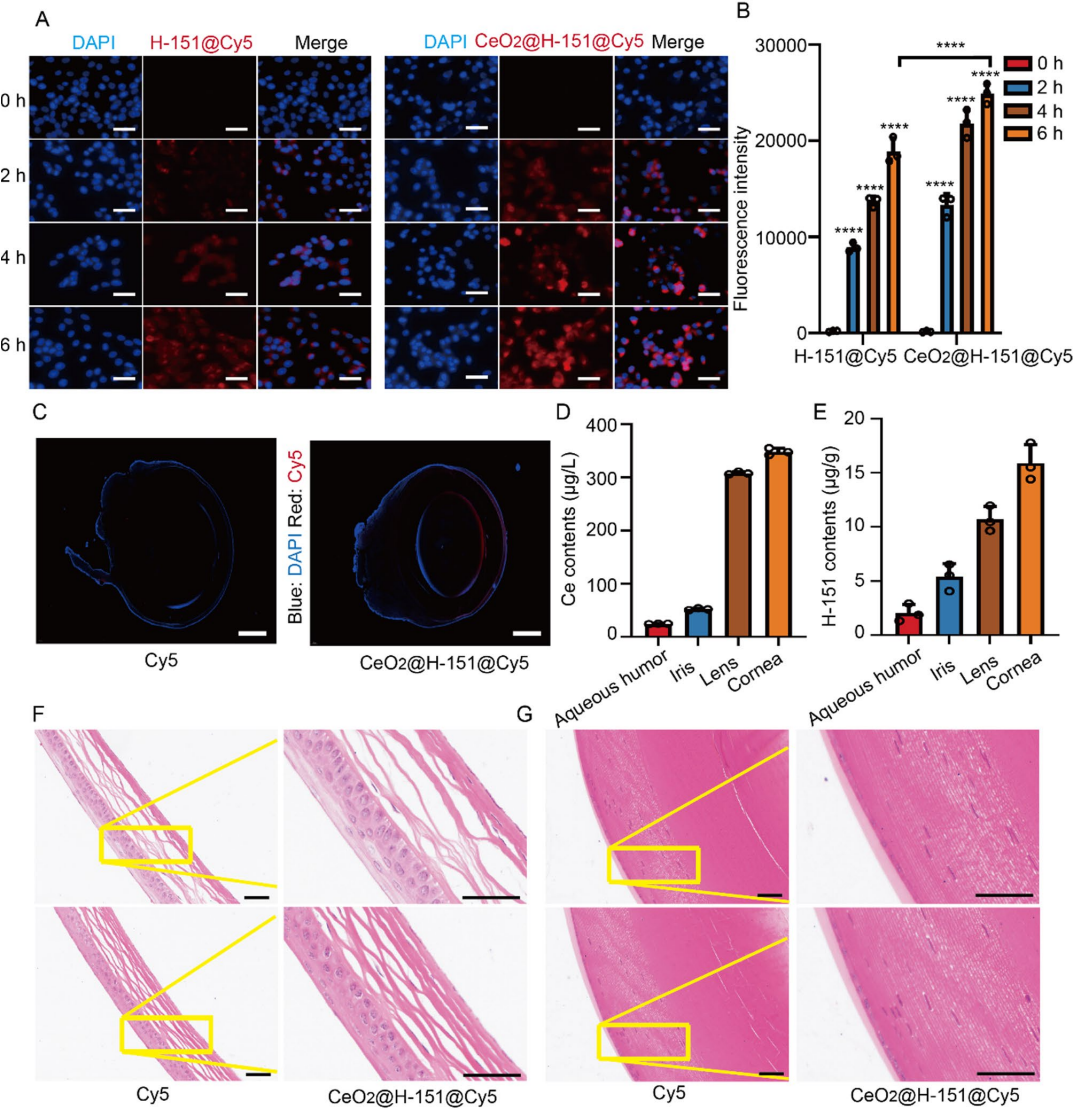
*Ex vitro* uptake and in vivo retention and distribution of CeO_2_@H-151 nanozymes. (**A**-**B**) CLSM images and statistical analysis of HLECB3 cells after incubation with H-151@Cy5 and Cy5-labeled CeO_2_@H-151 nanozymes for 0, 2, 4, 6 h (*n* = 3). Scale bar: 50 μm. (**C**) Fluorescence images of Cy5 and CeO_2_@H-151@Cy5 after topical eye drops for 35 days by ex vivo tissue. Scale bar: 400 μm. (**D**) The amount of Ce from CeO_2_@H-151@Cy5 in the mice eye including aqueous humor, iris, lens, cornea and retina (*n* = 3). (**E**) The amount of H-151 from CeO_2_@H-151@Cy5 in the mice eye including aqueous humor, iris, lens, cornea and retina (*n* = 3). (**F**) Representative histological images of cornea and lens (**G**) after topical eye drops with Cy5 and CeO_2_@H-151@Cy5 for 35 days. Scale bar: 50 μm. ****P < 0.0001

The biocompatibility of nanozymes is important for ocular drug delivery and therapies [37]. In vitro cytotoxicity assessment revealed that CeO_2_@H-151 nanozymes exhibited no significant toxicity on LECs across a wide concentration range (5–100 µg/mL) and different incubation periods from 6 h to 72 h (Fig. S6A-B), indicating their excellent biocompatibility and suitability for further investigation. To evaluate cellular uptake efficiency, H-151 and CeO_2_@H-151 were labeled with Cy5 for fluorescence tracking. After 2 h incubation, confocal laser scanning microscopy (CLSM) analysis revealed both H-151@Cy5 and CeO_2_@H-151@Cy5 (red) initiated endocytosis into the LECs (Fig. 4A-B). Subsequently, the CeO_2_@H-151@Cy5 group exhibited a time-dependent increase in red fluorescence intensity, demonstrating significantly stronger red fluorescence compared to H-151@Cy5 alone, which indicated the enhanced cellular endocytosis capability of the nanozyme.

#### Ex vivo and in vivo evaluation of CeO₂@H-151 nanozymes delivery and safety

We further evaluated the corneal retention of Cy5-labeled CeO_2_@H-151 nanozymes following topical administration using ex vivo ocular tissue section analysis. As shown in Fig. 4C, the red fluorescence intensity of Cy5@CeO_2_@H-151 was significantly higher in the cornea compared to H-151@Cy5, indicating that the CeO_2_-based nanoformulation enhances corneal penetration. This property is critical for delivering therapeutic agents into the eye and prevent IR-induced lens damage. Besides, to assess the biodistribution and biosafety of CeO_2_@H-151 nanozymes in vivo, we quantified Ce accumulation in ocular tissues using inductively coupled plasma optical emission spectrometry (ICP-OES) after 35 days of topical administration. Figure 4D demonstrated substantial Ce retention in the cornea, with detectable diffusion into the aqueous humor and lens, suggesting efficient transcorneal permeation. Consistent with these findings, ultraviolet-visible (UV-Vis) spectroscopy indicated higher H-151 concentrations in the cornea and lens for the Cy5@CeO_2_@H-151 treatment group compared to H-151@Cy5 (Fig. 4E), further confirming the nanozyme’s enhanced ocular delivery efficiency. Next, we evaluated the safety of Cy5@CeO_2_@H-151 in vivo. As shown in Fig. S7, no significant hemolytic toxicity was observed according to the hemolysis ratios, indicating the biosafety of the Cy5@CeO_2_@H-151. Besides, hematoxylin-eosin (HE) staining examination (Fig. 4F-G) demonstrated that there was no obvious inflammation and pathological abnormalities in the cornea and lens, which suggested the good biocompatibility of CeO_2_@H-151 nanozymes and suitable for ocular application.

### In vitro antioxidative capacity of CeO_2_@H-151 nanozymes

**Fig. 5:**
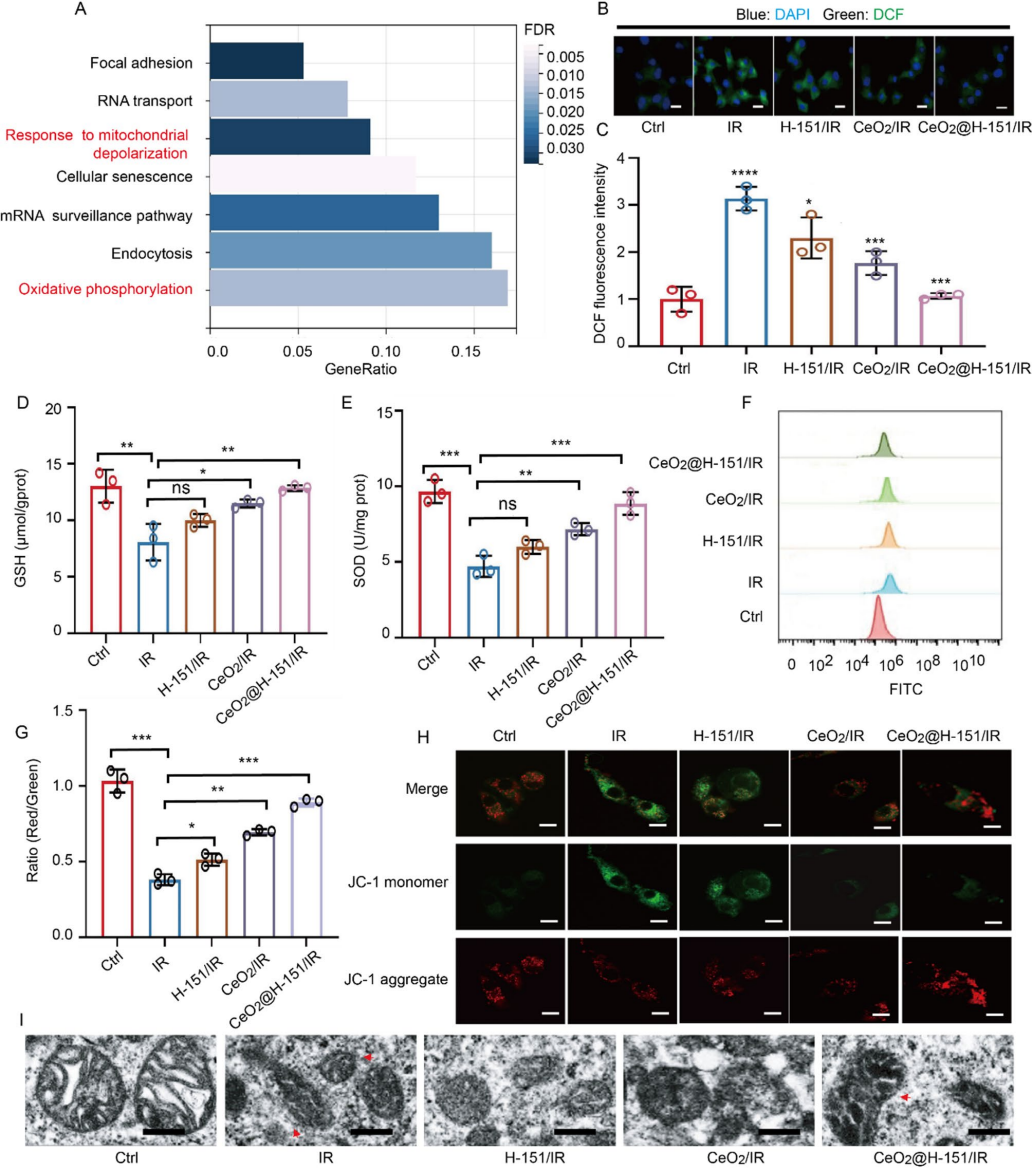
Antioxidative properties of CeO_2_@H-151 nanozymes in vitro. (**A**) KEGG enrichment analysis of differentially expressed genes between the control and IR-induced LECs. (**B**) Representative CLSM images of the DCFH-DA probe with treatments as indicated. Scale bar: 10 μm. (**C**) Statistics of DCF of MFI as indicated (*n* = 3). (**D**) Intracellular GSH and SOD (**E**) level of HLECB3 cells after treated with different formulas for 6 h (*n* = 3). (**F**) FCM analysis of the Mito-ROS probe with treatments as indicated. (**G**) Representative images and quantitative analysis (**H**) of JC-1 staining for each group of LECs. Red fluorescence represents JC-1 aggregates with high mitochondrial membrane potential (MMP) and green fluorescence represents JC-1 monomers with low MMP (*n* = 3). Scale bar: 10 μm. (**I**) TEM images of HLECB3 cells after different treatments. Scale bar: 250 nm. *P < 0.05, **P < 0.01, ***P < 0.001, ****P < 0.0001, and n.s., no significance

Redox imbalance plays a crucial role in the progression of IR-induced cataracts, where excessive ROS accumulation and compromised endogenous antioxidant defenses synergistically accelerate lens opacification [2]. Therefore, maintaining redox homeostasis and reducing oxidative stress could be effective strategies for preventing IR-induced cataract. We performed RNA sequencing (RNA-seq) on LECs subjected to IR exposure with or without CeO_2_@H-151 treatment. KEGG pathway analysis revealed significant enrichment of oxidative phosphorylation (*p* < 0.01) and mitochondrial depolarization response (*p* < 0.05) in the IR group, confirming IR-induced mitochondrial oxidative stress (Fig. 5A). Notably, these pathways were markedly suppressed by CeO_2_@H-151 treatment, suggesting its protective role in maintaining mitochondrial function. Besides, the volcano exhibited that CeO_2_@H-151 administration significantly downregulated oxidative stress-related genes including *Foxo4*, *Fosb* and *Jun* (Fig. S8), which are associated with ROS-mediated damage and inflammation [38]. Conversely, it upregulated cytoprotective genes *Cbx4* and *Bmp2*, known to enhance cellular stress resistance and tissue repair [39]. These findings indicated that CeO_2_@H-151 nanozymes mitigate IR-induced damage by (1) scavenging excess ROS, (2) preserving mitochondrial integrity, and (3) modulating stress-responsive gene networks.

To assess the antioxidant capacity of CeO_2_@H-151 nanozymes in IR-induced LECs damage, we first measured intracellular ROS levels using the 2′,7′-dichlorodihydrofluorescein diacetate (DCFH-DA) assay. As shown in Fig. 5B-C, CeO_2_@H-151 nanozymes demonstrated significantly greater ROS-scavenging ability compared to either H-151 or CeO_2_ alone in IR-exposed LECs. This enhanced antioxidant effect likely stems from the synergistic effect of H-151’s anti-inflammatory properties and CeO_2_’s redox-modulating capacity, which may break the vicious cycle between oxidative stress and inflammation. Flow cytometry (FCM) analysis further confirmed the superior ROS-reducing capability of CeO_2_@H-151 nanozymes (Fig. 5D). As illustrated in Fig. 5E-F, CeO_2_@H-151 nanozymes significantly restored the levels of superoxide dismutase (SOD) and glutathione (GSH) in IR-exposed LECs, both of which are crucial for maintaining redox homeostasis.

Due to mitochondria are the primary source of ROS generation, we investigated mitochondrial function using JC-1 probes to measure mitochondrial membrane potential (*ΔΨm*). CeO_2_@H-151 nanozymes effectively maintained *ΔΨm* at normal levels following IR exposure, outperforming both H-151 and CeO_2_ alone (Fig. 5G-H). TEM images indicated that while IR exposure caused mitochondrial shrinkage and cristae disruption in LECs, CeO_2_@H-151 nanozyme treatment substantially preserved mitochondrial ultrastructure (Fig. 5I). These results demonstrated that CeO_2_@H-151 nanozymes effectively mitigate IR-induced oxidative damage and mitochondrial dysfunction in LECs, as evidenced by both transcriptomic analysis and functional assays.

### Inhibition of CeO_2_@H-151 nanozymes on cGAS/STING-mediated inflammation stimulated by cytosolic DsDNA in mitochondria

**Fig. 6:**
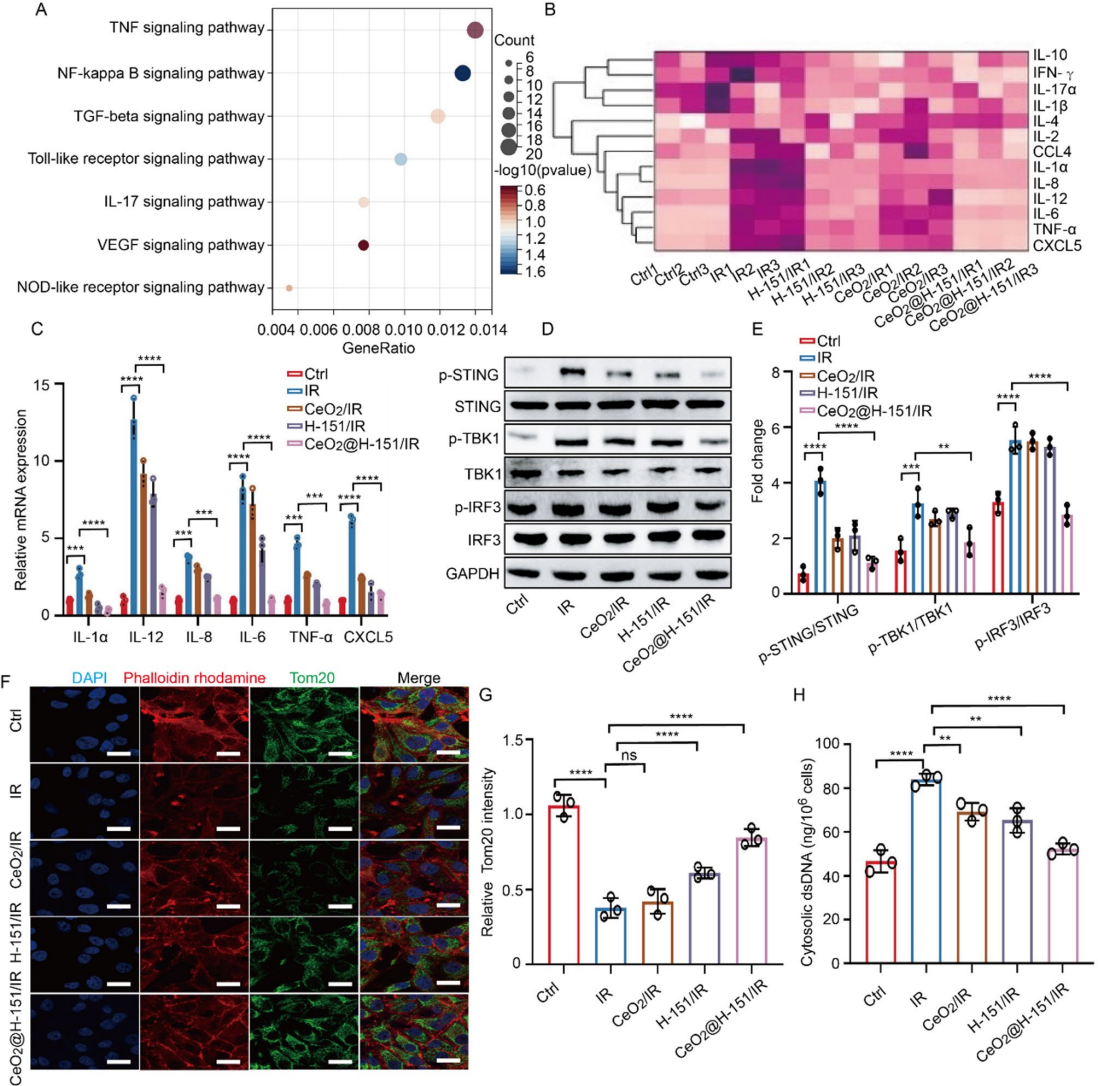
Anti-inflammatory properties of CeO_2_@H-151 nanozymes in vitro. (**A**) KEGG enrichment analysis of representative inflammatory cytokine pathways in the control and IR-induced LECs. (**B**) Heatmap of ELISA results under IR induction and indicated treatments (*n* = 3). (**C**) IL-1α, IL-12, IL-8, IL-6, TNFα and CXCL5 were determined by RT-qPCR (*n* = 4). (**D**-**E**) WB and quantitative analysis of p-STING/STING, p-TBK1/TBK1 and p-IRF3/IRF3 proteins after IR stimulation with indicated treatments. (**F**-**G**) IF staining and quantitative analysis of Tom20 (green) in LECs with treatments as indicated (*n* = 3). (**H**) Cytosolic dsDNA levels in HLECB3 cells after 24 h treatments as indicated (*n* = 3). **P < 0.01, ***P < 0.001, ****P < 0.0001, and n.s., no significance

Ionizing radiation (IR)-induced oxidative stress activates inflammatory cascades, including the upregulation of pro-inflammatory cytokines such as TNF-α and interleukins, exacerbating cellular damage and dysfunction [40]. Previous studies have demonstrated that IR exposure markedly elevates pro-inflammatory markers in lens epithelial cells (LECs), suggesting that suppressing IR-triggered inflammation may prevent cataract [41, 42]. Similarly, we also found that IR induction activated several important inflammatory pathways through RNA-seq data (Fig. 6A). Due to the anti-inflammatory properties of H-151, we evaluated the inhibitory effects of CeO_2_@H-151 nanozymes on IR-induced inflammation in LECs. ELISA assay showed that both H-151 and CeO_2_ nanoparticles downregulated key inflammatory mediators, including IL-1α, IL-12, IL-6, IL-8, TNF-α, and CXCL5, while CeO_2_@H-151 nanozymes almost inhibited cytokine production (Fig. 6B). Quantitative real-time PCR (RT-qPCR) further confirmed that while H-151 and CeO_2_ partially mitigated inflammation, CeO_2_@H-151 nanozymes exhibited superior suppression of IR-induced inflammatory responses in LECs (Fig. 6C). Since oxidative stress activates the cGAS-STING inflammasome, we assessed the impact of CeO_2_@H-151 nanozymes on this pathway. Western blot analysis demonstrated that CeO_2_@H-151 nanozymes significantly attenuated IR-induced phosphorylation of cGAS and STING, as well as downstream TBK1 and IRF3, outperforming H-151 or CeO_2_ alone (Fig. 6D). These findings indicated that CeO_2_@H-151 nanozymes effectively suppress cGAS/STING-mediated inflammatory signaling.

Considering the interaction between mitochondrial dysfunction and inflammation [43], we investigated whether CeO_2_@H-151 nanozymes could reduce IR-induced mitochondrial damage. Immunofluorescence staining of TOMM20 (Fig. 6E-F), a mitochondrial marker, indicated that CeO_2_@H-151 nanozymes robustly restored mitochondrial morphology and function compared to partial recovery observed with H-151 or CeO_2_ treatment alone. Inflammatory responses often provoke mitochondrial DNA (mtDNA) leakage into the cytosol, activating cGAS and perpetuating inflammation via STING-dependent interferon production. As shown in Fig. 6G, we can observe CeO_2_@H-151 nanozymes markedly reduce IR-induced cytosolic dsDNA accumulation, while H-151 or CeO_2_ alone showed limited efficacy. These results suggest that CeO_2_@H-151 nanozymes mitigate inflammation by preventing mtDNA-driven cGAS-STING activation. Our findings demonstrated that CeO_2_@H-151 nanozymes exert potent anti-inflammatory effects by (1) scavenging pro-inflammatory cytokines, (2) inhibiting cGAS-STING signaling, and (3) preserving mitochondrial integrity to prevent cytosolic dsDNA release. This multi-modal action positions CeO_2_@H-151 nanozymes as a promising therapeutic strategy for IR-induced inflammatory damage.

### CeO_2_@H-151 nanozymes exert anti-inflammatory effects through oxidation modification of the C172 site in PRDX2

**Fig. 7:**
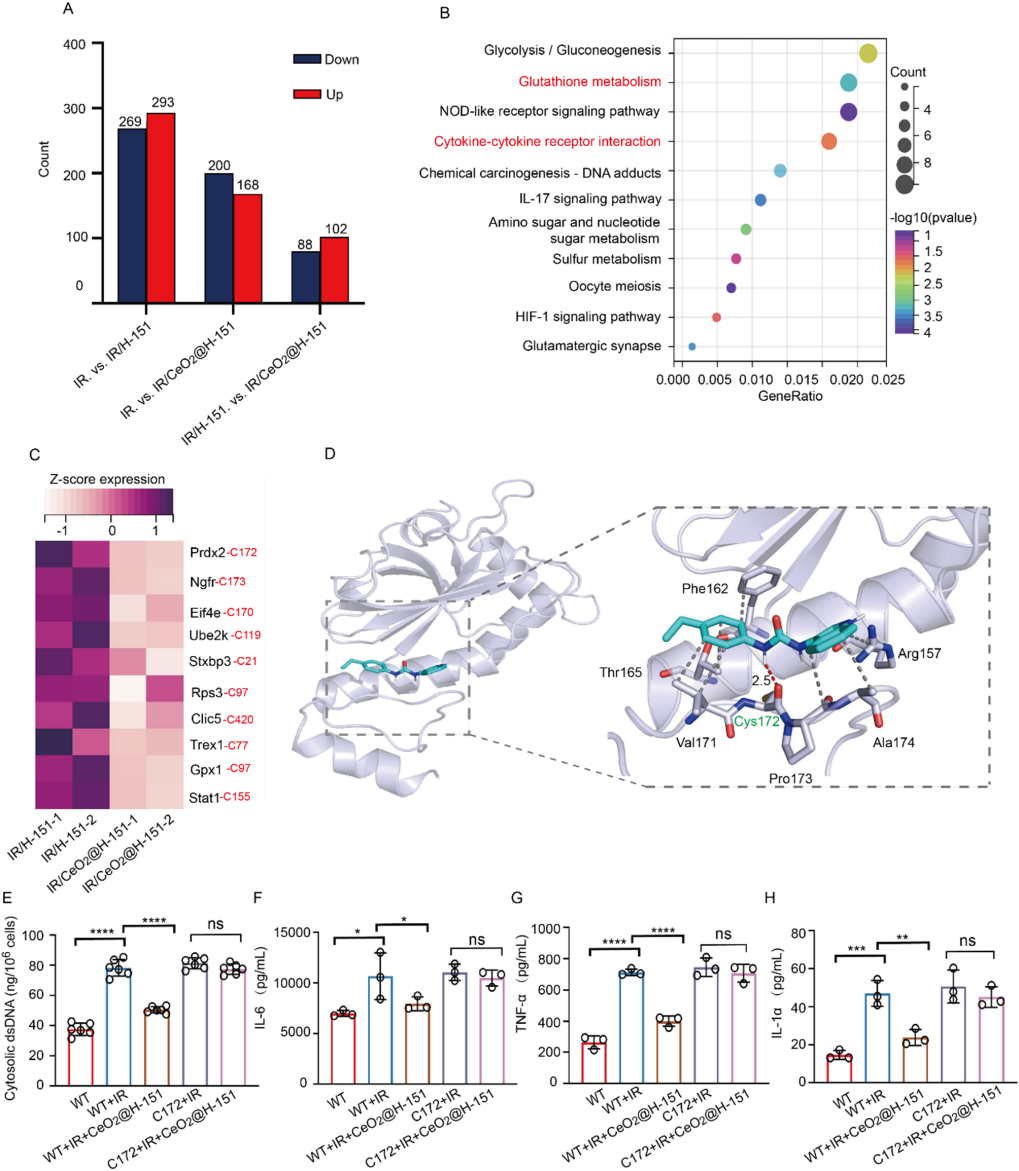
Anti-inflammatory effects of CeO_2_@H-151 nanozymes through oxidation modification of the C172 site in PRDX2. (**A**) The number of significantly differentially oxidation modification sites under treatments as indicated. (**B**) The KEGG function enrichment analysis after H-151 and CeO_2_@H-151 treatment. (**C**) Heatmap analysis of differential proteins modifications abundance in enriched glutathione metabolism, cytokine-cytokine receptor interaction, negative regulation of inflammatory response and antioxidant activity pathways (*n* = 2). (**D**) Stereo view of the interaction between PRDX2 and H-151. Red and grey dotted lines denote hydrogen bonds and hydrophobic interaction. H-151 is displayed in stick colored by cyan, while the PRDX2 target protein is shown in grey. (**E**) Cytosolic dsDNA levels in HLECB3 cells after 24 h treatments as indicated (*n* = 3). (**F**-**H**) ELISA analysis of TNF-α, IL-6, IL-1α under indicated treatments (*n* = 3). *P < 0.05, **P < 0.01, ***P < 0.001, ****P < 0.0001, and n.s., no significance

Cataracts are characterized by the aggregation and denaturation of crystallin proteins, leading to vision impairment [44–46]. Oxidative modifications in proteins promote crystallin proteins aggregation, leading to insoluble deposits [47]. To indicate the underlying mechanism of how CeO_2_@H-151 nanozymes affects inflammatory, we conducted total oxidative modifications proteomics in IR-irradiated mice. Principal component analysis (PCA) displayed distinct protein modification shift (Fig. S9), with CeO_2_@H-151 treatment demonstrating superior oxidative protection (368 modified sites) compared to H-151 alone (562 sites) (Fig. 7A). Comparative analysis identified 190 significantly modulated oxidative modification sites (Fig. S10), with KEGG pathway analysis highlighting concurrent enhancement of glutathione metabolism and suppression of cytokine-receptor interactions (Fig. 7B). GO analysis demonstrated the activation of antioxidant pathways and inhibition of inflammatory regulators (Fig. S11), aligning with our previous findings on nanozyme bioactivity in vitro. As shown in Fig. 7C, CeO_2_@H-151 treatment substantially attenuated oxidative modifications in key stress-response proteins, including PRDX2, GPX1, and TREX1. Furthermore, molecular docking was used to assess the interactions between H-151 and the proteins, revealing the strong binding of H-151 to PRDX2 (docking score: 5.185) was superior to other targets (Fig. S12-14, Table S1). Notably, PRDX2 is an antioxidant enzyme primarily responsible for eliminating hydrogen peroxide and toxic ROS in cells, thereby reducing oxidative stress and inhibiting the activation of inflammation-related signaling pathways such as NF-κB [48, 49]. Structural analysis further identified C172 as the critical redox-active site mediating PRDX2 interaction through hydrogen bonding (Fig. 7D and Fig. S14). Functional validation demonstrated that anti-inflammatory effect of CeO_2_@H-151’s was achieved by reducing dsDNA accumulation (Fig. 7E) and suppressing cytokine secretion including IL-6, IL-1α, and TNF-α (Fig. 7F-H), which were dependent on PRDX2-C172 and ineffective in C172-mutant cells. In addition, RT-qPCR assay was consistent with the above ELISA results (Fig. S15). These findings demonstrated that CeO_2_@H-151 nanozymes exert the therapeutic effects via site-specific oxidative modulation of PRDX2 at C172, thereby simultaneously maintaining protein homeostasis, enhancing antioxidant capacity and suppressing inflammatory cascades.

### CeO_2_@H-151 nanozymes prevents IR-induced cataracts by inhibiting Crystallin aggregation in vivo

**Fig. 8:**
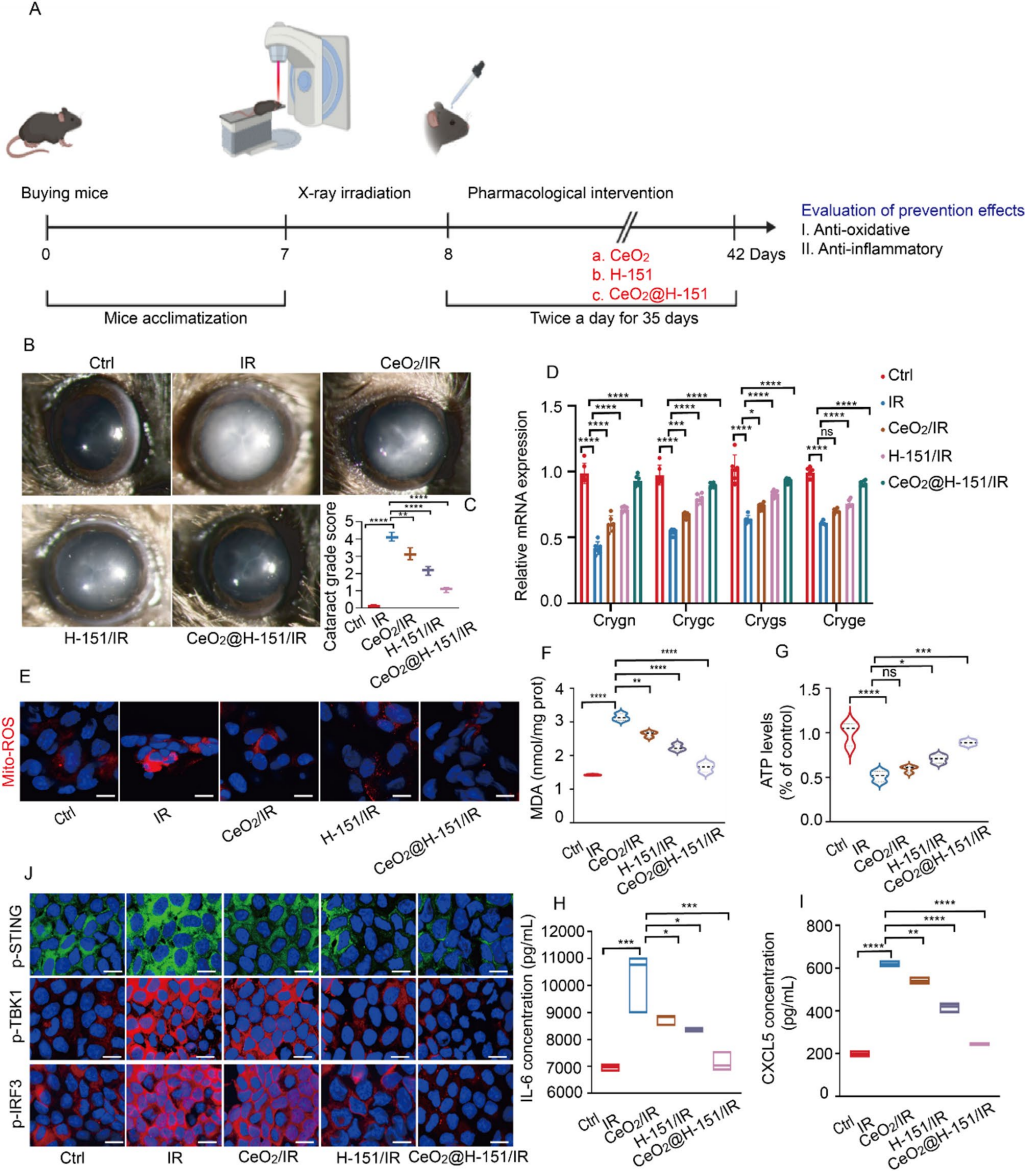
In vivo prevention effect of CeO_2_@H-151 nanozymes against IR-induced cataracts. (**A**) Schematic of in vivo mice IR-induced cataract model establishment, indicated treatment, and following assessment. (**B**) Observation of the lens opacity through a slit lamp. (**C**) Bermudez scoring according to a 0 − 4-point system (*n* = 3). (**D**) RT-qPCR analysis of Crygn, Crygc, Crygs, Cryge mRNA expression of mice lens with indicated treatments in consecutive days (*n* = 3). (**E**) Representative CLSM images of the Mito-ROS probe after the mice lens flat-mount with treatments as indicated. Scale bar: 10 μm. (**F**) MDA and ATP (**G**) levels of mice lens under treatments as indicated in consecutive days (*n* = 3). (**H**) ELISA analysis of IL-6 and CXCL5 (**I**) in the mice aqueous humor under IR induction and indicated treatments (*n* = 3). (**J**) Mice lens flat-mount IF staining of p-STING (green), p-TBK1 (red) and p-IRF3 (green) after IR induction with indicated treatments. Scale bar: 10 μm. *P < 0.05, **P < 0.01, ***P < 0.001, ****P < 0.0001, and n.s., no significance

We further evaluated the therapeutic efficacy of CeO_2_@H-151 nanozymes in preventing IR-induced cataracts in vivo. Following a single 16 Gy X-ray exposure, mice were topically administrated with H-151, CeO_2_ nanozymes, and CeO_2_@H-151 nanozymes for 35 days, respectively, and the long-term protective effects were evaluated (Fig. 8A). As shown in Fig. 8B, cataract was quantified using the Bermudez Grading system (0–4) and monitored via slit-lamp imaging. IR-exposure triggered lens opacification, culminating in mature cataracts (Grade 4) in untreated mice. In contrast, CeO_2_ and H-151 monotherapy attenuated opacification to Grades 3 and 2, respectively, while CeO_2_@H-151 nanozymes Markedly preserved lens transparency, with scored Grade 1 (Fig. 8C). These findings demonstrated the superior efficacy of CeO_2_@H-151 in preventing IR-induced cataract.

Crystallin α, β, and γ are essential for maintaining lens transparency and refractive function through their highly ordered structural organization [50]. Disruption of crystallin homeostasis through protein misfolding or aggregation represents a key driving factor in cataractogenesis [51–53]. As shown in Fig. S16, RNA-seq analysis revealed that IR exposure significantly downregulated key crystallin genes (*Crygn*,* Crygc*,* Crygs*,* Cryge*) in murine lenses. Similarly, quantitative RT-qPCR indicated that CeO_2_@H-151 nanozymes effectively restored expression of these crystallins post-IR (Fig. 8D), suggesting preservation of lens optical properties. Due to the correlation between mitochondrial dysfunction and cataract formation, we also performed co-enrichment analysis of IR-induced DEGs with mitochondrial gene sets, identifying mt-ND5, mt-CYTB, and mt-ATP6 as key targets (Fig. S17A), and CeO_2_@H-151 nanozymes significantly suppressed IR-induced overexpression of these mitochondrial genes (Fig. S17B) As shown in Fig. 8E, CeO_2_@H-151 nanozymes obviously decreased the Mito-ROS concentration compared to the CeO_2_ and H-151 treatments after IR exposure. Biochemical analyses further revealed that CeO_2_@H-151 nanozymes markedly decreased MDA levels (Fig. 8F) and restored ATP production compare to the H-151 and CeO_2_ nanozymes (Fig. 8G), confirming protection against lipid peroxidation and energy metabolism collapse. Supplementary ELISA showed a significant decrease in inflammatory cytokines (IL-6, CXCL5) caused by IR elevation in aqueous humor (Fig. 8H-I). Immunofluorescence and Western blot assays exhibited that CeO_2_@H-151 nanozymes could significantly inhibit the phosphorylation levels of STING, TBK1 and IRF3 compared to that of the treatment of H-151 and CeO_2_ after IR exposure (Fig. 8J and Fig. S18, S19A-B). Collectively, these results established that CeO_2_@H-151 nanozymes prevent IR-induced cataracts through the synergic effects of anti-oxidative and anti-inflammatory.

## Conclusion

In summary, this study illustrated the pivotal role of the cGAS-STING pathway in IR-induced cataract pathogenesis and proposed a novel nanozyme-based targeted therapeutic strategy. We first established that IR activates the cGAS-STING pathway, driving oxidative stress and inflammatory cytokine release, thereby disrupting lens homeostasis. Herein, we developed the CeO_2_@H-151 nanozymes, and demonstrated that the CeO_2_ nanozyme effectively mitigates mitochondrial dysfunction through robust ROS scavenging. On the other hand, H-151 could inhibit the cGAS-STING pathway via cytosolic dsDNA suppression and prevent oxidative modification of the PRDX2 protein. Following local ocular administration in murine models, CeO_2_@H-151 nanozymes displayed superior prevention of IR-induced lens opacification through synergistic antioxidant, anti-inflammatory, and protein homeostasis-preserving effects. These findings not only validate cGAS-STING inhibition as a viable prevent strategy against radiation-induced cataract but also highlight the clinical translational potential of this nanozyme-based approach for ophthalmic therapy.

## Supplementary Information

Supplementary Material 1.

Supplementary Material 1

## Data Availability

No datasets were generated or analysed during the current study.
