# Supplementary material for: Drug loaded cerium oxide nanozymes prevent radiation-Induced cataracts via suppressing the cGAS-STING pathway
Source: J Nanobiotechnology. 2025 Oct 10;23:657. doi: 10.1186/s12951-025-03706-2 (PMC12514853; doi:10.1186/s12951-025-03706-2)

**Drug Loaded Cerium Oxide Nanozymes Prevent Radiation-Induced Cataracts via Suppressing the cGAS-STING Pathway**

Qifang Chen ^a,†^, Peilin Gu ^a,†^, Hongwei Li ^b,†^, Xuemei Liu ^a^, Ting Liu ^a,^*, Qin Ouyang ^b,^*, Dong Liu ^c,^*, Chongyi Li ^a,^*

^a^Department of Ophthalmology, Daping Hospital, Army Medical University, Chongqing 400042, China.

^b^Department of Pharmaceutical Chemistry, Army Medical University, Chongqing 400038, China.

^c^State Key Laboratory of Ophthalmology, Zhongshan Ophthalmic Center, Sun Yat-sen University, Guangzhou 510060, China.

*Correspondence: liuting0727@tmmu.edu.cn (T. Liu); ouyangq@tmmu.edu.cn (Q. Ouyang); liud99@mail.sysu.edu.cn (D. Liu); lichongyi@tmmu.edu.cn (C. Li);

^†^Q. Chen, P. Gu and H. Li contributed equally to this work.


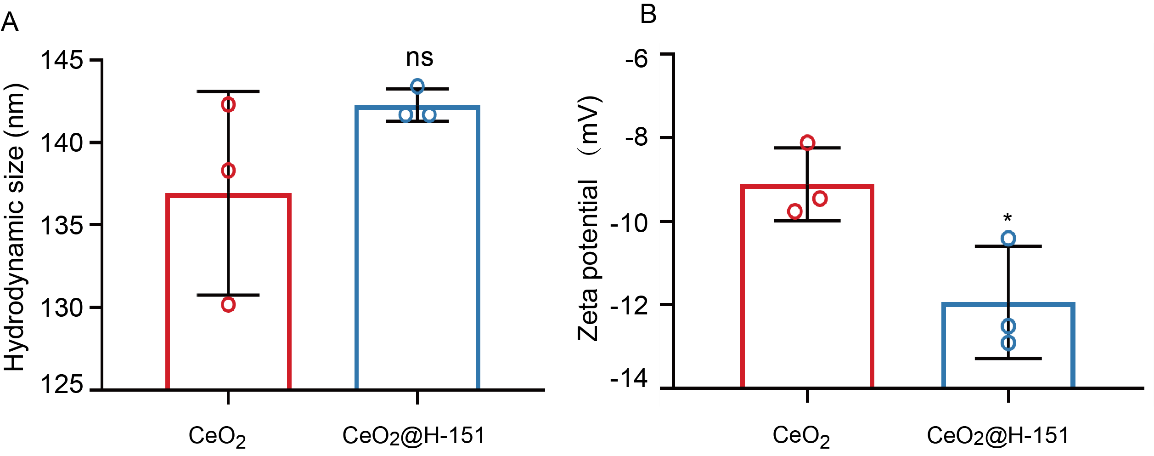


**Fig. S1.** **Characterization of the prepared CeO_2_@H-151 nanozymes.** (A) DLS of CeO_2_ and CeO_2_@H-151 nanozymes (n = 3). (B) Zeta potentials of CeO_2_ and CeO_2_@H-151 nanozymes (n = 3). **P*<0.05, and n.s., no significance.


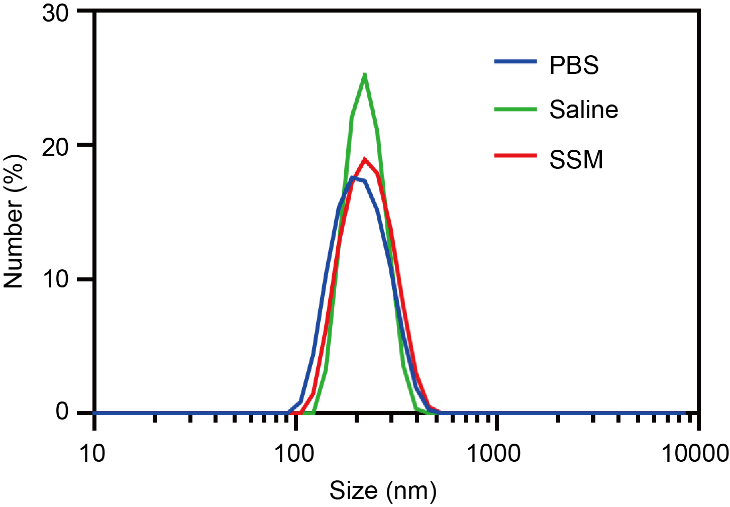


**Fig. S2.** Size of CeO_2_@H-151 in phosphate-buffered saline (PBS), saline (0.9% sodium chloride (NaCl)), and serum-supplemented medium (SSM) from DLS.


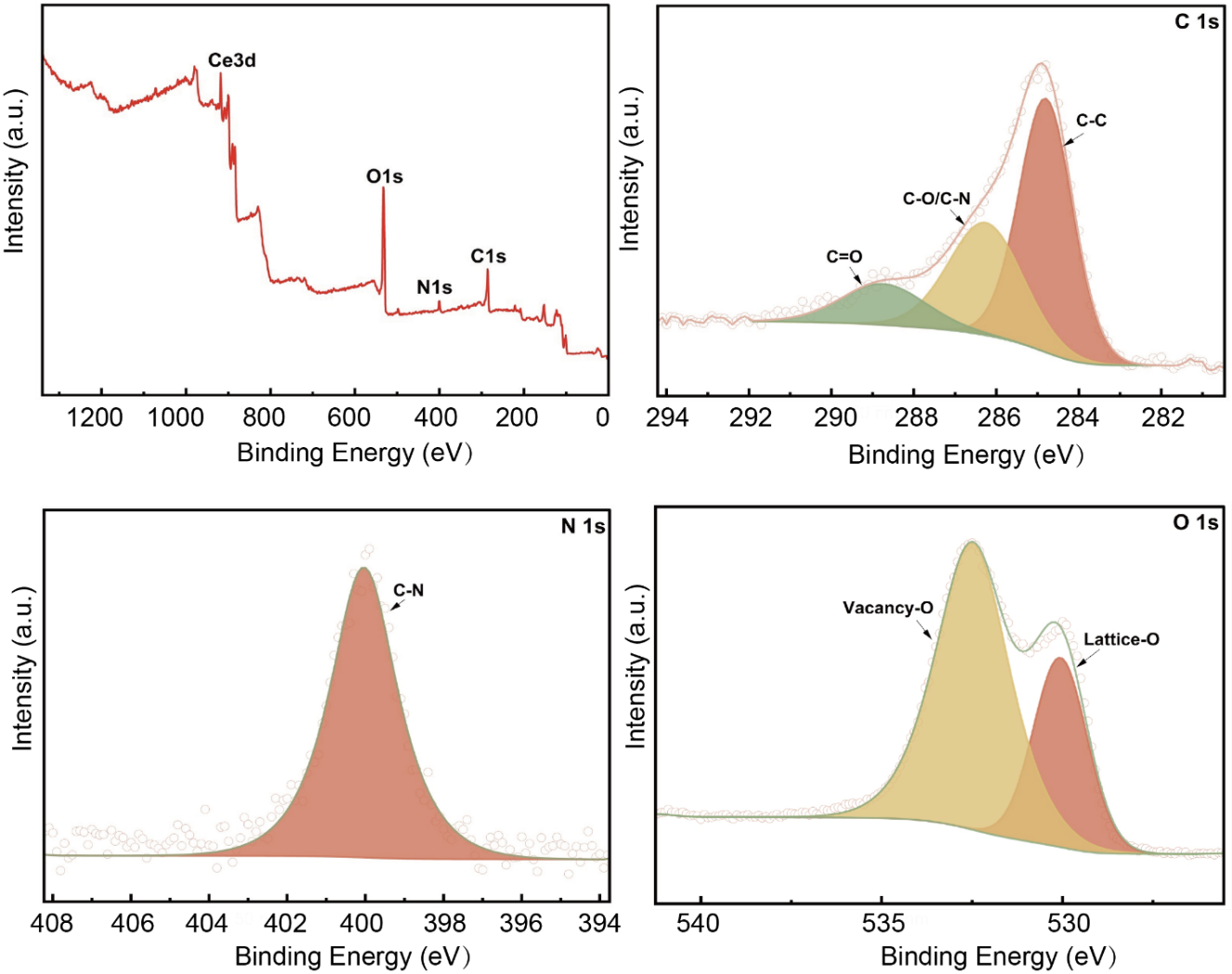


**Fig. S3.** XPS analysis of CeO_2_@H-151 nanozymes.


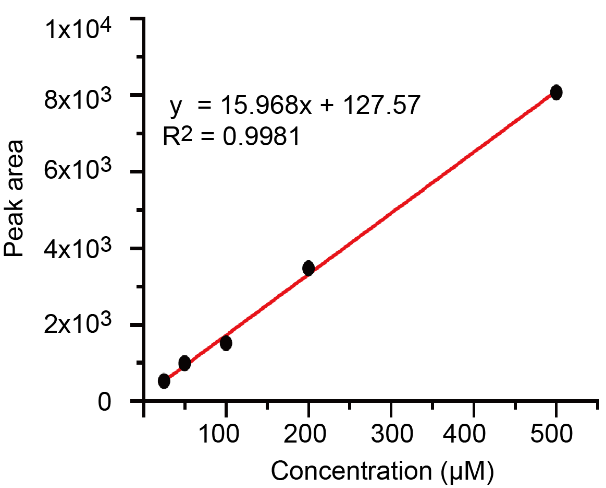


**Fig. S4.** The calibration curve of H-151 by HPLC.


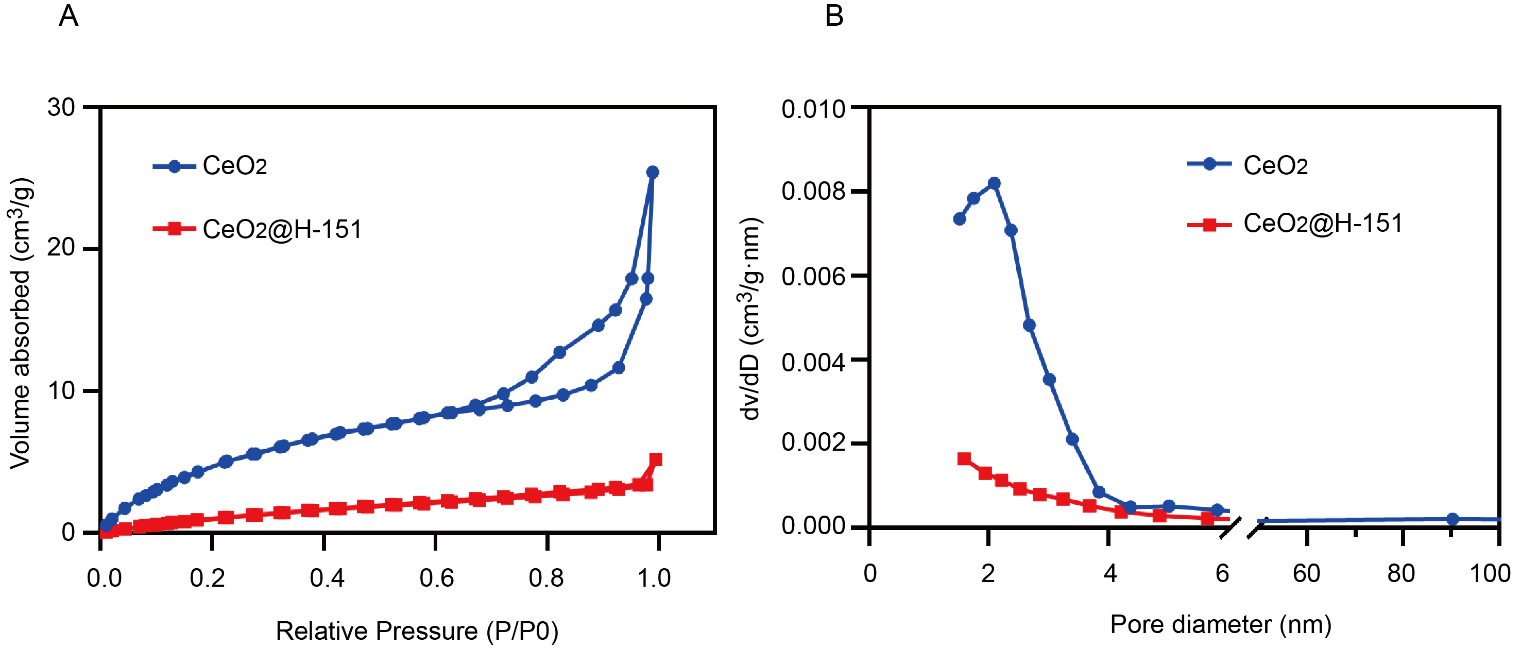


**Fig. S5. BET analysis of CeO_2_ and CeO_2_/H-151.** (A) N_2_ adsorption isotherms of CeO_2_ and CeO_2_/H-151. (B) Pore size distributions of CeO_2_ and CeO_2_/H-151.


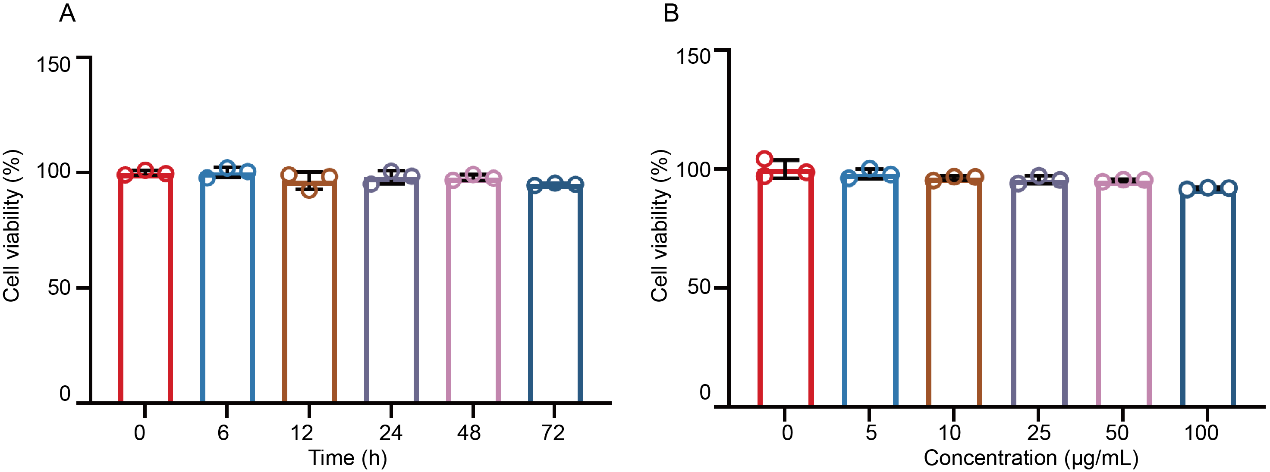


**Fig. S6. Cytotoxicity assessment of CeO_2_@H-151 nanozymes.** (A) Cell viability of HLECB3 cells treated with CeO_2_@H-151 nanozymes at 100 μg/mL for different durations. (B) Cell viability of HLECB3 cells treated with different concentrations of CeO_2_@H-151 nanozymes for 72 h.


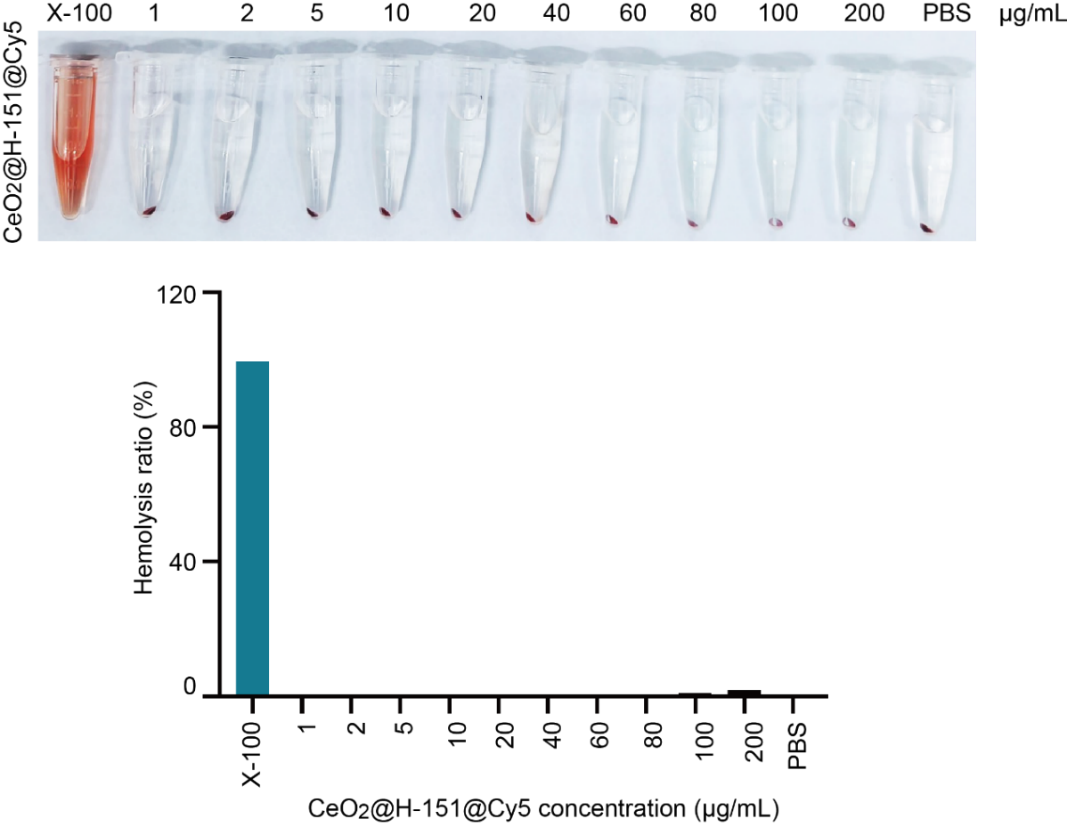


**Fig. S7.** The photos of hemolysis assay treated with CeO_2_@H-151@Cy5 at different concentrations and hemolysis ratios of CeO_2_@H-151@Cy5.


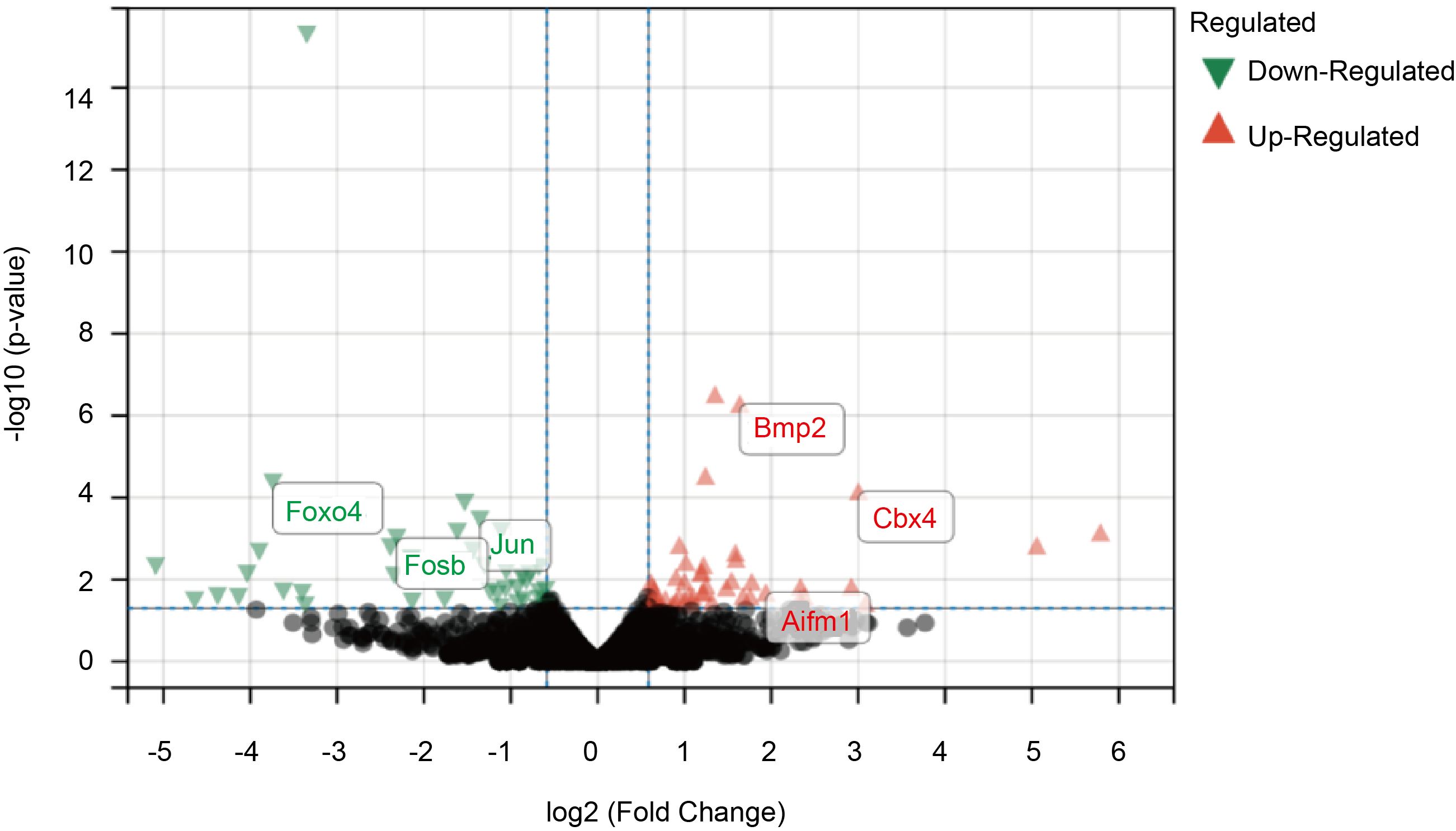


**Fig. S8.** Volcano plot of gene expression in IR exposure with or without CeO₂@H-151 treatment.


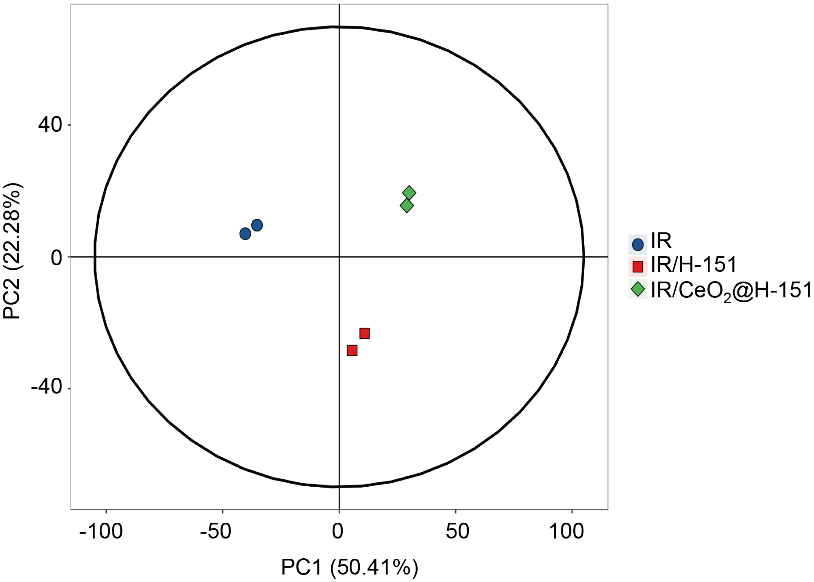


**Fig. S9.** PCA analysis after IR induction with H-151 and CeO_2_@H-151 nanozymes treatments.


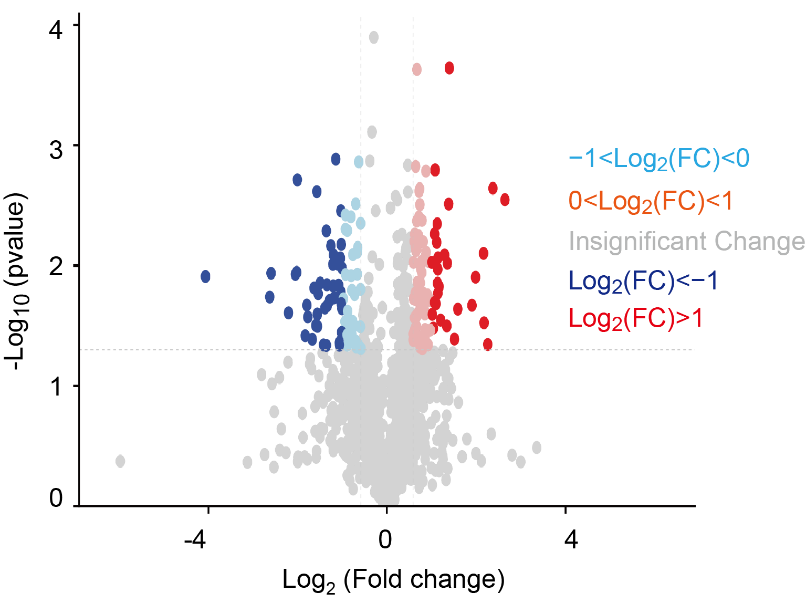


**Fig. S10.** Volcano plot of protein modifications abundance in H-151 and CeO_2_@H-151 groups.


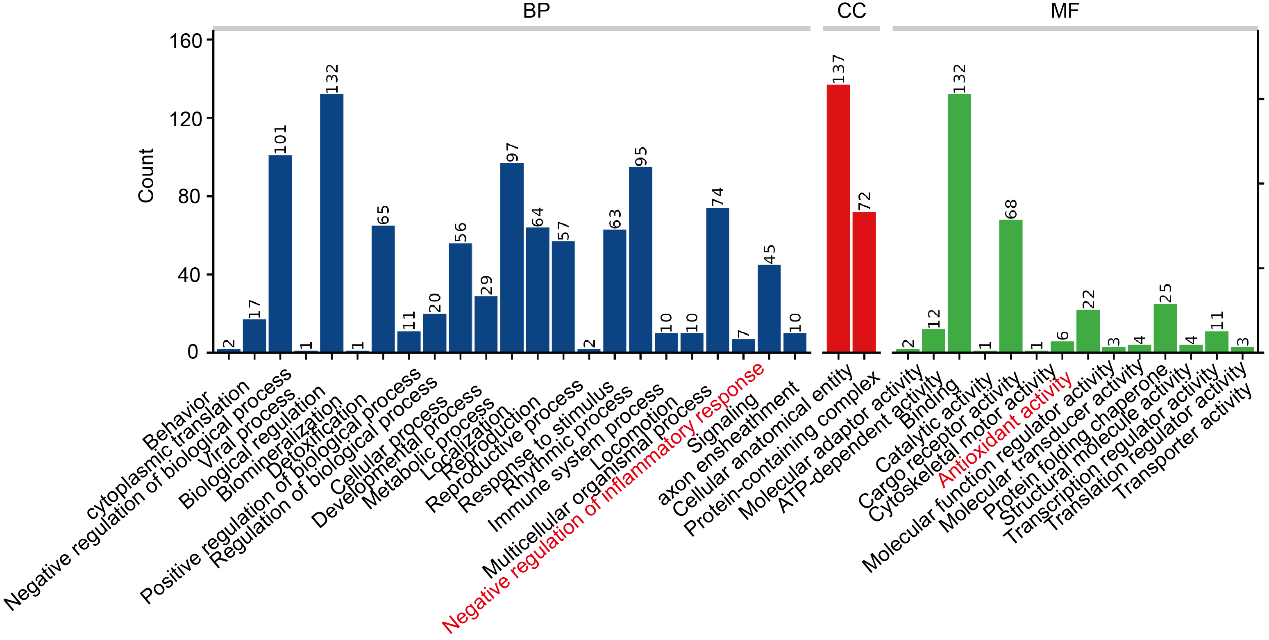


**Fig. S11.** The GO analysis after IR/H-151 and IR/ CeO_2_@H-151 treatment.


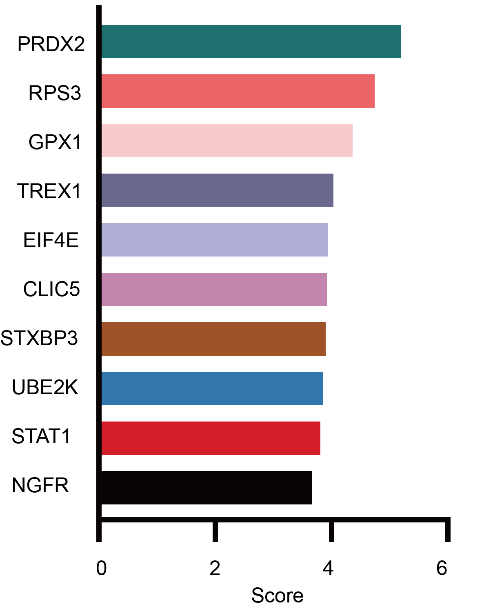


**Fig. S12.** Average molecular docking score of 10 differential proteins by Sybyl and AutoDock Vina.


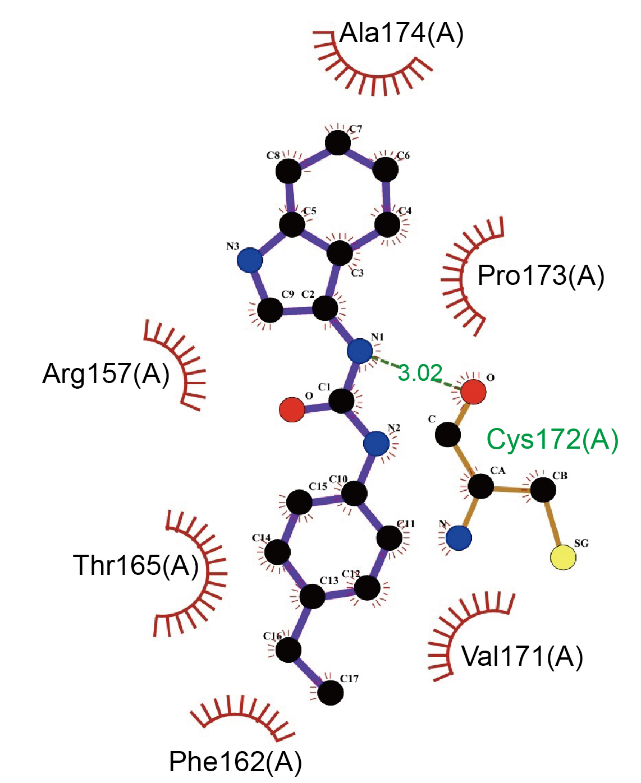


**Fig. S13.** The 2D visualization of the interaction between PRDX2 and H-151.


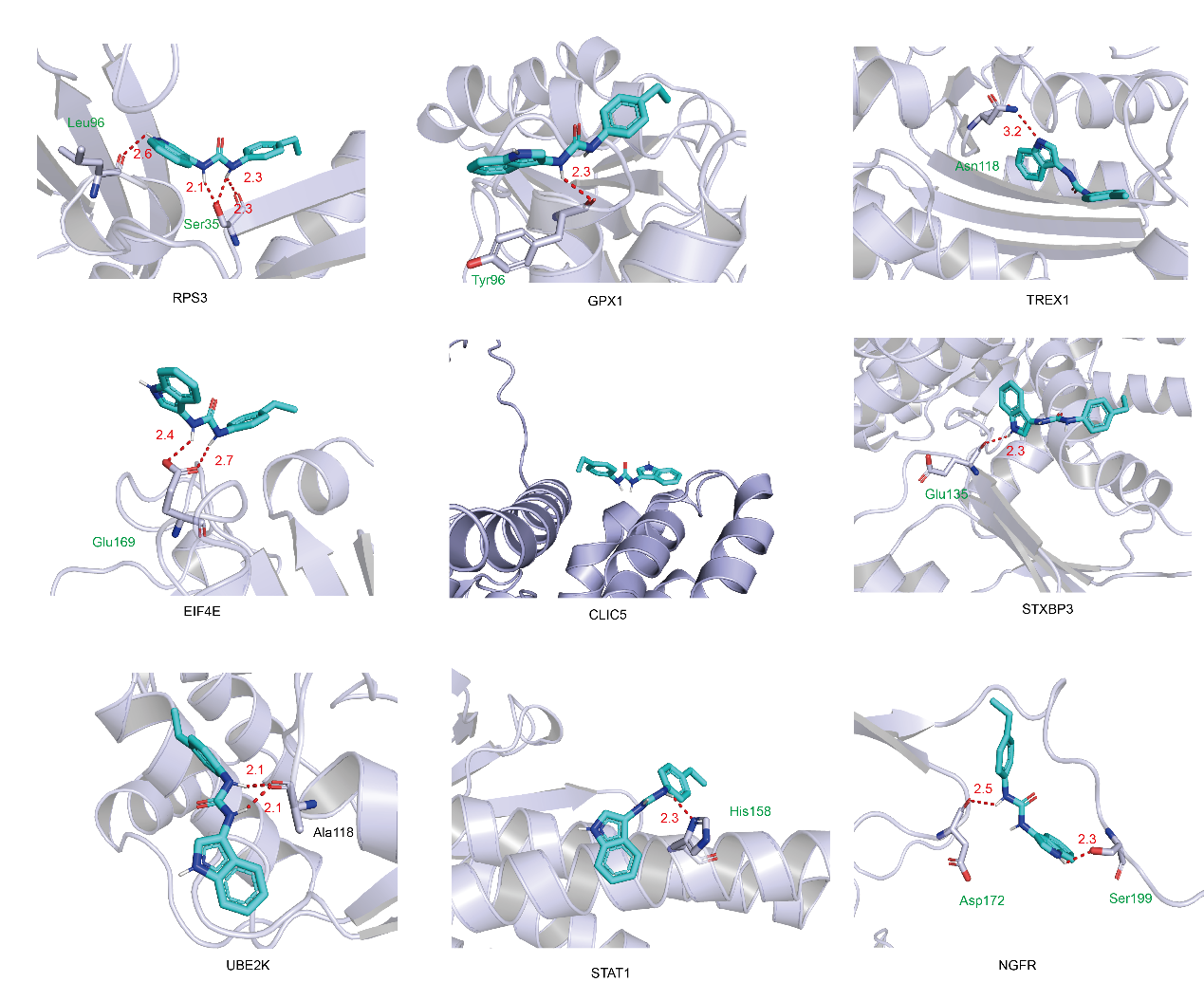


**Fig. S14.** Two- and three-dimensional mapping of the binding sites between H-151 and the other nine target protein. H-151 is displayed in green, while the TREX1 target protein is shown in blue.


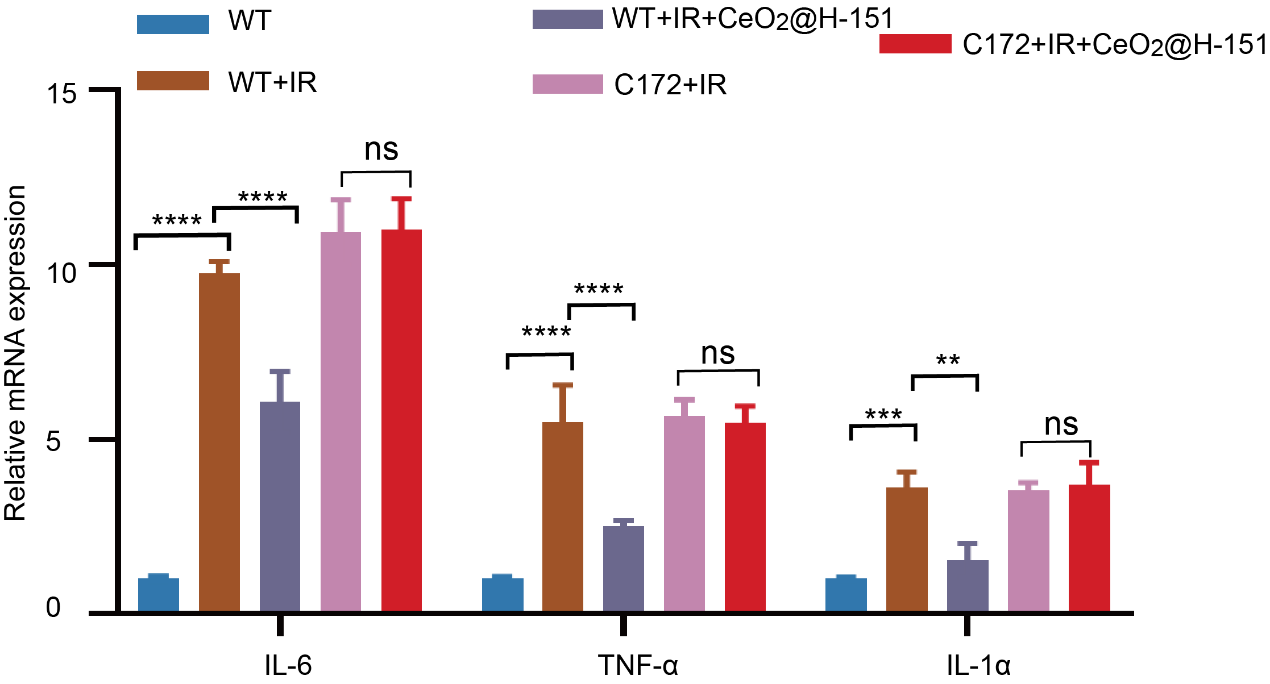


**Fig. S15.** RT-qPCR analysis of IL-1α, IL-6 and TNFα with treatments as indicated (n=3). ***P* < 0.01, ****P* < 0.001, *****P* < 0.0001, and n.s., no significance.


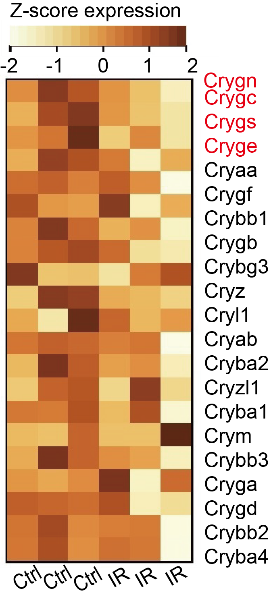


**Fig. S16.** Heatmap of RNA-seq data of the control and IR-induced LECs.


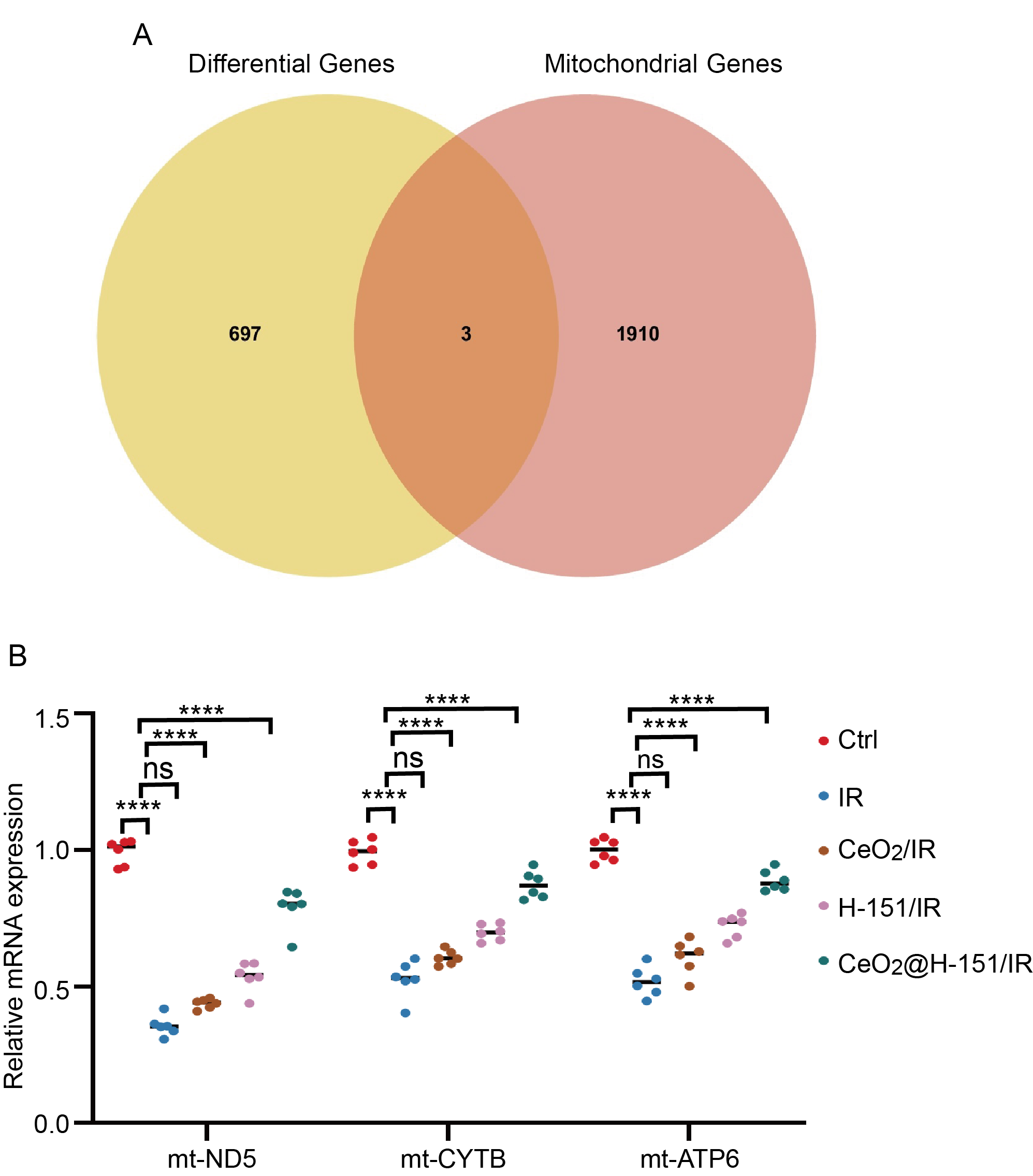


**Fig. S17. The regulation of mitochondrial function genes by CeO_2_@H-151.** (A) Venn diagram showing shared three genes under differential genes of IR-induced LECs and mitochondrial genes. (B) RT-qPCR analysis of mt-ND5, mt-CYTB, mt-ATP6 mRNA expression of mice lens of different groups in consecutive days (n=3). *****P* < 0.0001, and n.s., no significance.


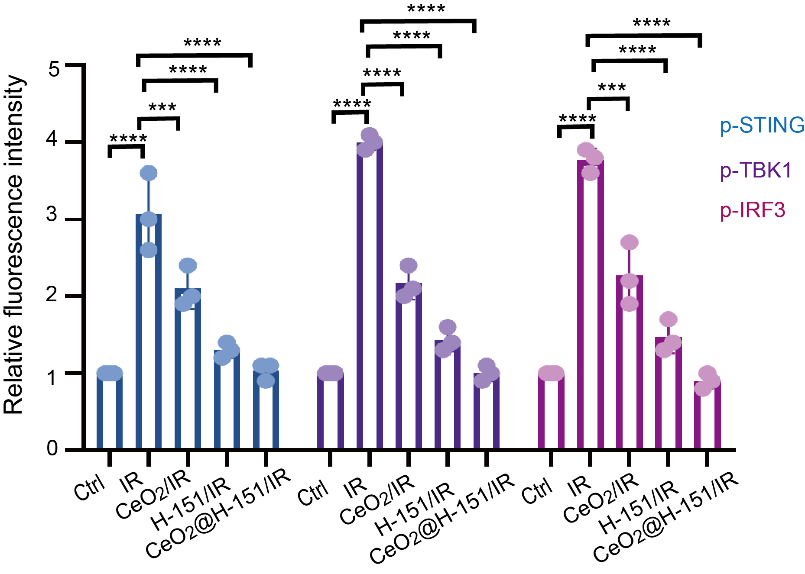


**Fig. S18.** Quantitative analysis of IF staining in p-STING, p-TBK1 and p-IRF3 proteins with treatments as indicated (n=3). ****P* < 0.001, *****P* < 0.0001.


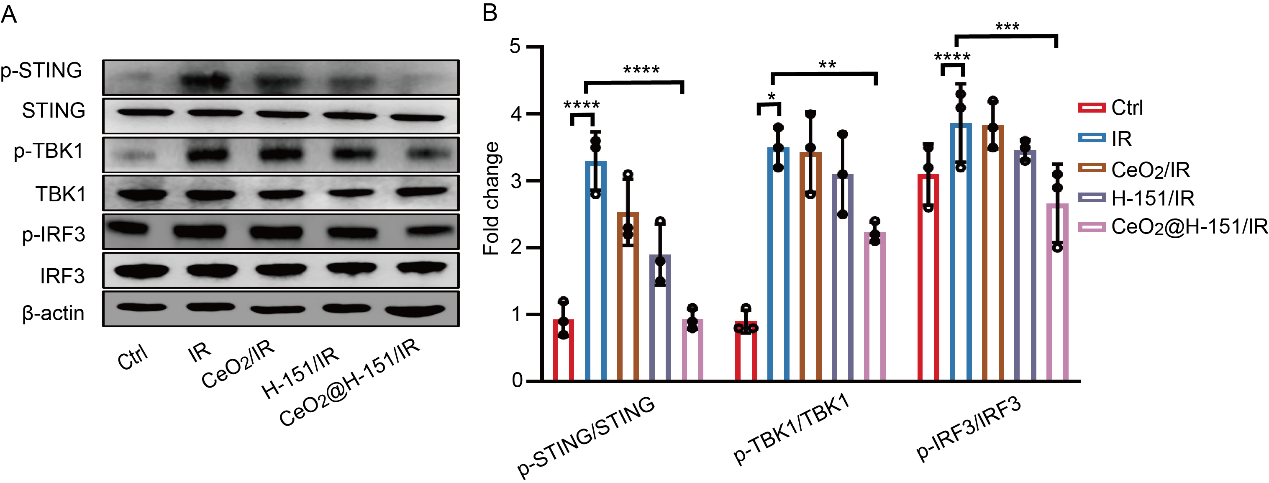


**Fig. S19. Inhibition of STING pathway and TBK1-IRF3 downstream pathways by CeO_2_@H-151.** (A-B) WB and quantitative analysis of p-STING, p-TBK1 and p-IRF3 proteins in the mice lens with treatments as indicated (n=3). **P* < 0.05, ***P* < 0.01, ****P* < 0.001, *****P* < 0.0001, and n.s., no significance.

**Table S1.** Molecular docking score of 10 differential proteins by Sybyl and AutoDock Vina.


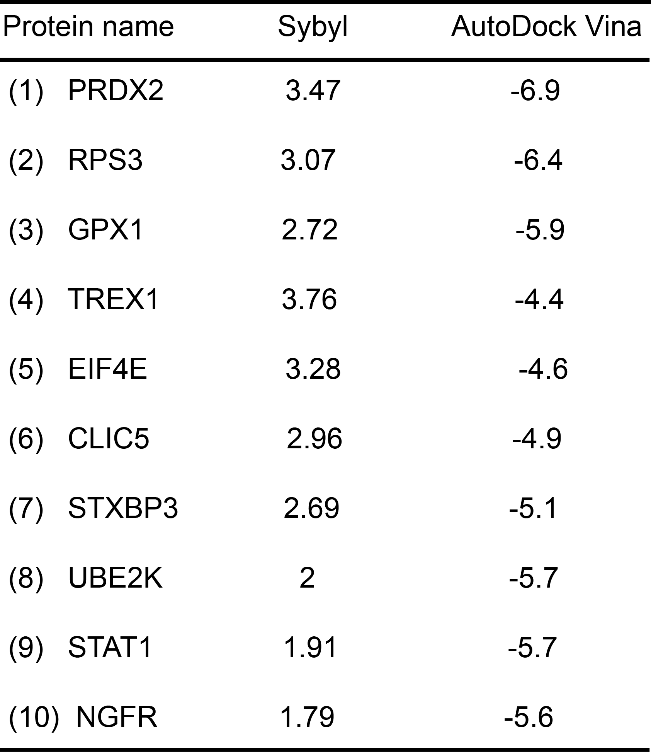

Supplement: Supplementary file 2 — Supplementary Material 1 [file 12951_2025_3706_MOESM2_ESM.docx]
